# Crystal structures of [μ_2_-(*R_a_*,*S_a_*,3a*R*,7a*R*)-1,3-bis­(2,7-di­cyclo­hexyl­naphthalen-1-yl)octa­hydro-1*H*-benzo[*d*]imidazolidin-2-yl­idene]chlorido­(η^4^-1,5-cyclo­octa­diene)iridium di­chloro­methane monosolvate and [μ_2_-(*S_a_*,*S_a_*,3a*R*,7a*R*)-1,3-bis­(2,7-di­cyclo­hexyl­naphthalen-1-yl)octa­hydro-1*H*-benzo[*d*]imidazolidin-2-yl­idene]chlorido­(η^4^-1,5-cyclo­octa­diene)iridium

**DOI:** 10.1107/S2056989020011603

**Published:** 2020-09-04

**Authors:** Stephen A. Moggach, Brian W. Skelton, Daven J. Foster

**Affiliations:** a Centre for Microscopy, Characterisation and Analysis, University of Western Australia, 35 Stirling Highway, 6009 Perth, Western Australia, Australia; b Department of Chemistry, School of Molecular Sciences, University of Western Australia, M310, 35 Stirling Highway, 6009 Perth, Western Australia, Australia

**Keywords:** crystal structure, *N*-heterocyclic carbene, iridium, naphth­yl, cyclo­octa­diene

## Abstract

The title compounds, [Ir(C_51_H_64_N_2_)Cl(C_8_H_12_)]·CH_2_Cl_2_, (I), and [Ir(C_51_H_64_N_2_)Cl(C_8_H_12_)], (II), represent the first two examples of hexa­hydro­benzo­imidazole-based *N*-heterocyclic carbene (NHC) iridium complexes. The diastereomeric complexes differing only in their axial chirality, which could be separated *via* column chromatography, show noticeable differences in their ^1^H NMR spectra. Compound (I) crystallizes in the monoclinic system (*P*2_1_) with two independent complexes and two half-occupied di­chloro­methane mol­ecules in the asymmetric unit, while compound (II) crystallizes in the ortho­rhom­bic system (*P*2_1_2_1_2_1_) with one complex in the asymmetric unit. The fused five-membered *N*-heterocycles of NHCs show unusually high backbone torsion angles of −34.1 (5) and −30.9 (5)° for (I) and −31.5 (7)° for (II), but the Ir—C_carbene_ bond lengths of 2.046 (6) and 2.021 (6) Å for (I) and 2.045 (8) Å for (II) present typical NHC—Ir bond lengths. The solvent mol­ecule in the crystal of (I) was found to be highly disordered and its contribution to the scattering was masked using the solvent-masking routine smtbx.mask in *OLEX2* [Dolomanov *et al.* (2009 ▸). *J. Appl. Cryst.* **42**, 339–341]. The solvent contribution is not included in the reported chemical formula and other crystal data.

## Abstract 2

The title compounds, [Ir(C_51_H_64_N_2_)(C_8_H_12_)Cl]·CH_2_Cl_2_ and [Ir(C_51_H_64_N_2_)(C_8_H_12_)Cl], represent the first two examples of hexa­hydro­benzo­imidazole-based *N*-heterocyclic carbene (NHC) iridium complexes. The diastereomeric complexes differing only in their axial chirality, which could be separated *via* column chromatography, show noticeable differences in their ^1^H NMR spectra.

## Chemical context   

**Figure 1:**
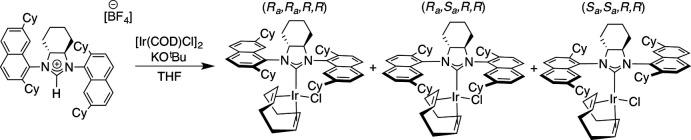
Reaction scheme for the synthesis of the title compounds.

The use of *N*-heterocyclic carbenes (NHCs) as ancillary ligands for various metal complexes has been implemented extensively, resulting in the synthesis of many successful catalytic species. When enanti­opure catalysts are used, asymmetric transformations can be performed that result in enantio-enriched synthetic products. In terms of chiral NHC ligand design, substituents on the NHC that are too distal to the coordination sphere of the metal centre generally result in low enantio-selectivities for such catalysts. Some NHCs featuring a fused, chiral cyclo­hexyl backbone (modelled on the salen-type or Trost-type ligands) have been reported and studied, but the catalytic capabilities of such complexes resulted in disappointing enantio-selection (Lee & Hartwig, 2001 ▸; Arao *et al.*, 2006 ▸; Luan *et al.*, 2008 ▸; Lai *et al.*, 2009 ▸). Using bulky naphthyl wingtips for the NHCs, axial chirality is generated due to hindrance of rotation around the C—N bonds. With implementation of an enanti­opure cyclo­hexyl backbone, three isomers are present in the mixture and are isolable *via* column chromatography as previously reported for similar Ir–NHC complexes (Gao *et al.*, 2020 ▸). Herein we report a new NHC ligand that uses bulky 2,7-di­cyclo­hexyl naphthyl wingtips on the NHC in the hope that it will result in iridium precatalysts that can successfully perform catalytic transformations with high enantio-selectivity (Fig. 1 ▸).
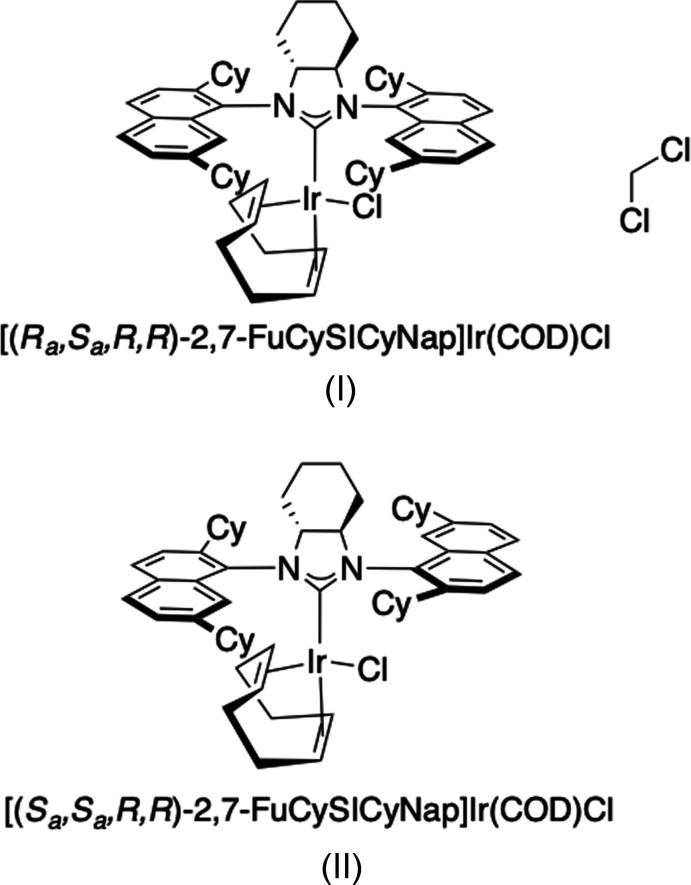


## Structural commentary   

**Figure 2:**
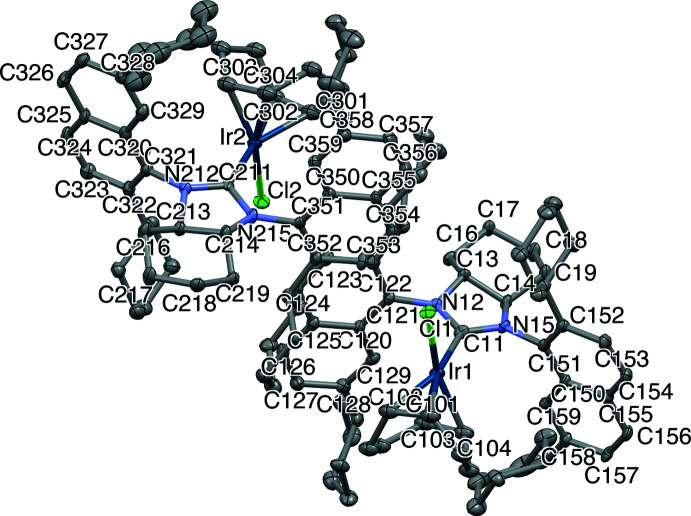
The mol­ecular structures of the title complex (I) with atom labelling. Labelling of selected aliphatic carbons has been omitted for clarity. Ellipsoids are drawn at the 50% probability level. H atoms have been omitted for clarity.

The mol­ecular structures of the title compounds, (I) and (II), are depicted in Figs. 2 ▸ and 3 ▸, respectively. The two compounds crystallized under the same conditions, where the complexes were dissolved in a minimum amount of di­chloro­methane (DCM) under an inert atmosphere and layered with pentane, which then slowly diffused into the DCM solution overnight resulting in the formation of yellow crystals. Compound (I) crystallizes in the monoclinic system (*P*2_1_) with two independent complexes and two half-occupied DCM mol­ecules in the asymmetric unit; the solvent DCM mol­ecules were masked in the refinement due to high disordering. Compound (II) crystallizes in the ortho­rhom­bic system (*P*2_1_2_1_2_1_) with one complex in the asymmetric unit.

The overall geometry of the complexes is square planar. The cyclo­hexyl rings in the fused *N*-heterocyclic ring systems of (I) and (II) adopt a chair conformation, which lead to the distortion of the 5-membered imidazolidine ring. The torsion angles corresponding to N12—C13—C14—N15 are −34.1 (5) and −30.9 (5)° for (I) and −31.5 (7)° for (II). The Ir—C_carbene_ bond lengths are 2.046 (6) and 2.021 (6) Å for (I) and 2.045 (8) Å for (II). With respect to other complexes of general formula (NHC)Ir(COD)Cl, where COD is cyclo­octa­diene, these bond lengths are quite similar to 2.041 (3) Å in (SIMes)Ir(COD)Cl, 2.049 (5) Å in (SIPr)Ir(COD)Cl (Kelly *et al.*, 2008 ▸), 2.034 (1) Å in (*anti*-2-SICyNap)Ir(COD)Cl, 2.052 (5) Å in (*anti*-2,7-SICyNap)Ir(COD)Cl (Sipos *et al.*, 2016 ▸), 2.034 (3) Å in (*anti*-2-SICyOctNap)Ir(COD)Cl and 2.053 (5) Å [(*R_a_,R_a_,S,S*)-DiPh-2,7-SICyNap]Ir(COD)Cl (Gao *et al.*, 2020 ▸). Whilst being shorter than the values of 2.072 (6) and 2.055 (6) Å for two independent mol­ecules of (2-SIMorNap)Ir(COD)Cl (Ou *et al.*, 2017 ▸), the bonds in the title compounds are slightly longer than in [(*R_a_,S_a_,S,S*)-DiPh-2-SICyNap]Ir(COD)Cl [2.028 (7) Å; Gao *et al.*, 2020 ▸]. DiPh (di-phen­yl) and FuCy (fused cyclo­hex­yl) refer to the backbone of the NHC, and SI (saturated imidazolium) refers to the NHC type. Mes (2,4,6-trimethyl phen­yl), Pr (2,6-diisopropyl phen­yl), CyNap (cyclo­hexyl 1-naphthalene), CyOctNaph (cyclo­octyl 1-naphthalene) and MorNap (morpholinyl 1-naphthalene) refer to the side-chain substitution.

Saturated achiral or racemic NHC–Ir–COD complexes without a fused second ring appear to have a greater degree of flexibility and show clearly smaller distortions within the five-membered *N*-heterocycle, with backbone torsion angles ranging from 1.6° for (*anti*-2-SICyNap)Ir(COD)Cl to 19.5° for (SIPr)Ir(COD)Cl. For enanti­opure complexes, [(*R_a_,R_a_,S,S*)-DiPh-2-SICyNap]Ir(COD)Cl has a torsion angle of 9.4° and [(*R_a_,R_a_,S,S*)-DiPh-2,7-SICyNap]Ir(COD)Cl has 11.9°, with a small range in angles of different mol­ecules in the same crystal (Δ0.3°), revealing that increased bulk on the backbone appears to increase the rigidity of the NHC ring.

## Supra­molecular features   

**Figure 4:**
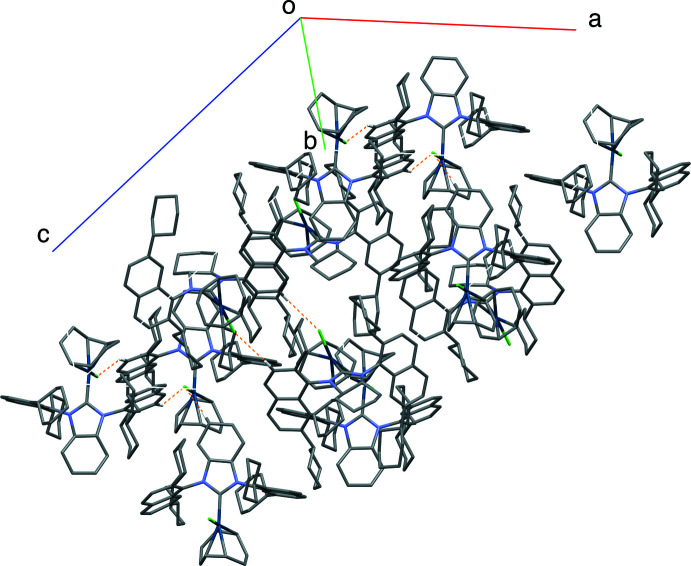
Mol­ecular packing in the crystal of (I) with C—H⋯Cl inter­actions highlighted in orange.

In the crystal of (I), the complex mol­ecules are stacked in a column along the *b* axis *via* weak C—H⋯Cl inter­actions (Table 1 ▸ and Fig. 4 ▸). In comparison, (II) has no obvious inter­actions between mol­ecules.

## Database survey   

The only other report of a crystallographically characterized fused cyclo­hexyl NHC complex is (FuCySIMes)Rh(COD)Cl (CSD Refcode BUNRIM; Lai *et al.*, 2009 ▸). The backbone torsion angle in the NHC was 32.0° (average of two independent mol­ecules), slightly larger than those found in the title complexes. Selected examples of other crystallographically characterized saturated NHC-iridium-COD complexes with aromatic wingtips include (see *Structural commentary* for abbreviations) (SIMes)Ir(COD)py[PF_6_] (XIDLAX; Lee, Jiang *et al.*, 2001 ▸), (SIMes)Ir(COD)Cl (QIWPAO), (SIPr)Ir(COD)Cl (QIWPIW) (Kelly *et al.*, 2008 ▸), [K](OCO)Ir(COD) [OCO = 1,3-di(2-hy­droxy-5-*tert*-butyl­phen­yl)imidazolyl; USOZIM; Weinberg *et al.*, 2010 ▸], chiral (*anti*-2-SICyNap)Ir(COD)Cl (UMEGEA), (*anti*-2,7-SICyNap)Ir(COD)Cl (UMEGIE), (*anti*-2,7SICyNap)Ir(COD)[PF6] (UMEHIF) (Sipos *et al.*, 2016 ▸), (2-SIMorNap)Ir(COD)Cl (XARZUO; Ou *et al.* 2017 ▸) and (*anti*-2-SICyOctNap)Ir(COD)Cl (POWGAM; Gao *et al.*, 2020 ▸). Enanti­opure versions include [(*R_a_,R_a_,S,S*)-DiPh-2-SICyNap]Ir(COD)Cl (XUKYUA), [(*R_a_,R_a_,S,S*)-DiPh-2-SICyNap]Ir(COD)[PF_6_] (XUKYAG) and [(*R_a_,R_a_,S,S*)-DiPh-2,7-SICyNap]Ir(COD)Cl (XUKZIP) (Gao *et al.*, 2020 ▸).

## Synthesis and crystallization   

2,7-FuCySICyNap·HBF_4_ (300 mg, 0.38 mmol) was added to a solution of [Ir(COD)Cl]_2_ (138 mg, 0.18 mmol) in THF (8 mL) in a glovebox and was stirred at room temperature. KO^*t*^Bu (42 mg, 0.38 mmol) was then added and the yellow solution was stirred for 3 h. The yellow–brown solution was evaporated to dryness and the three diastereoisomers were separated *via* column chromatography (1.5 kg SiO_2_, diameter 5cm, 1:20 diethyl ether:hexa­ne) to afford the products as yellow powders after evaporation of the respective fractions under vacuum: yield 78 mg (21%) for (I), 40 mg (11%) for (II) and 101 mg (27%) for [(*R_a_,R_a_,R,R*)-2,7-FuCySICyNap]Ir(COD)Cl. The respective yellow powders were dissolved in DCM and layered with pentane to obtain yellow crystals of two of the three isomers, (I) and (II).

(I) ^1^H NMR (600 MHz, CD_2_Cl_2_): *δ* 7.97 (*s*, 1H, H^8^), 7.92–7.83 (*m*, 4H), 7.58 (*dd*, *J_a_* = 8.6 Hz, *J_b_* = 1.1 Hz, 2H), 7.50 (*s*, 1H, H^8^), 7.49–7.43 (*m*, 2H), 4.30–4.23 (*m*, 1H), 3.86–3.72 (*m*, 2H), 3.65–3.59 (*m*, 1H), 3.53–3.46 (*m*, 1H), 3.31–3.25 (*m*, 1H), 3.09–3.04 (*m*, 1H), 2.93–2.71 (*m*, 3H), 2.64 (*d*, *J* = 12.1 Hz, 1H), 2.48 (*d*, *J* = 11.6 Hz, 1H), 2.15–0.76 (*m*, 52H), 0.75–0.68 (*m*, 1H), 0.56–0.47 (*m*, 1H) ppm. ^13^C NMR (151 MHz, CD_2_Cl_2_): *δ* 214.2 (carbene), 146.4, 145.8, 145.5. 145.4, 133.5, 132.8, 132.2, 132.08, 132.05, 131.9, 128.9, 128.8, 128.7, 128.3, 125.5, 125.0, 124.6, 124.4, 122.3, 122.2, 84.7, 82.3, 71.6, 71.4, 54.5, 52.7, 46.2, 45.8, 40.7, 38.5, 37.8, 37.2, 35.7, 35.1, 34.3, 34.03, 34.01, 29.2, 29.1, 27.9, 27.80, 27.78, 27.4, 27.3, 27.2, 27.02, 27.00, 26.96, 26.9, 26.83, 26.79, 26.58, 26.55, 24.8, 24.5 ppm. Elemental analysis (%) calculated for C_59_H_76_N_2_IrCl: C 68.06, H 7.36, N 2.69. Found: C 68.21, H 7.56, N 2.82.

(II): ^1^H NMR (500 MHz, CD_2_Cl_2_): *δ* 8.22 (*s*, 1H), 7.89 (*d*, *J* = 8.7 Hz, 1H), 7.853 (*d*, *J* = 8.3 Hz, 1H), 7.848 (*d*, *J* = 8.3 Hz, 1H), 7.77 (*d*, *J* = 8.4 Hz, 1H), 7.61 (*d*, *J* = 8.7 Hz, 1H), 7.52 (*d*, *J* = 8.7 Hz, 1H), 7.44 (*s*, 1H), 7.43 (*d*, *J* = 8.2 Hz), 7.38 (*dd*, *J_a_* = 8.3 Hz; *J_b_* = 1.5 Hz, 1H), 4.21–4.11 (*m*, 1H), 4.10–3.98 (*m*, 2H), 3.78–3.64 (*m*, 2H), 3.36–3.23 (*m*, 2H), 2.90–2.73 (*m*, 3H), 2.49 (*d*, *J* = 11.9 Hz, 1H), 2.25 (*d*, *J* = 13.0 Hz, 1H), 2.12 (*d*, *J* = 12.6 Hz, 1H), 2.05–1.01 (*m*, 74H), 0.93–0.80 (*m*, 2H), 0.74–0.64 (*m*, 1H) ppm. ^13^C NMR (126 MHz, CD_2_Cl_2_): *δ* 212.1 (carbene), 146.41, 146.39, 144.6, 144.0, 132.8, 132.5, 132.1, 132.0, 131.89, 131.0, 129.0, 128.9, 128.5, 127.4, 126.6, 125., 125.7, 124.7, 124.3, 121.9 (aromatic carbons), 85.2, 74.1, 71.8, 70.1 (olefinic carbons), 50.0, 46.2, 45.9, 40.4, 39.0, 37.1, 36.8, 35.3, 34.7, 33.69, 33.68, 33.5, 33.3, 33.2, 29.9, 29.8, 28.9, 28.5, 27.9, 27.7, 27.6, 27.3, 27.2, 27.1, 26.9, 26.80, 26.79, 26.6, 26.4, 24.7, 24.6 ppm. Elemental analysis (%) calculated for C_59_H_76_N_2_IrCl: C 68.06, H 7.36, N 2.69. Found: C 68.55, H 7.61, N 2.54.

## Refinement   

Crystal data, data collection and structure refinement details are summarized in Table 2 ▸. For (I) the solvent mol­ecule was masked using the smtbx masking tool in *OLEX2* (Dolomanov *et al.*, 2009 ▸) due to diffuse electron density that could not be fitted using an atomistic model. The mask gave two void positions, of 428 and 414 Å^3^, and with 76.2 and 74.7 electrons, respectively. This equates to two half-occupied DCM mol­ecules. Hydrogen atoms were positioned geometrically (C—H = 0.95–1.00 Å) and refined using a riding model with *U*_iso_(H) = 1.2*U*_eq_(C).

## Supplementary Material

Crystal structure: contains datablock(s) I, II, global. DOI: 10.1107/S2056989020011603/is5546sup1.cif

Structure factors: contains datablock(s) I. DOI: 10.1107/S2056989020011603/is5546Isup2.hkl

Structure factors: contains datablock(s) II. DOI: 10.1107/S2056989020011603/is5546IIsup3.hkl

CCDC references: 1963571, 2025278

Additional supporting information:  crystallographic information; 3D view; checkCIF report

## Figures and Tables

**Figure 3:**
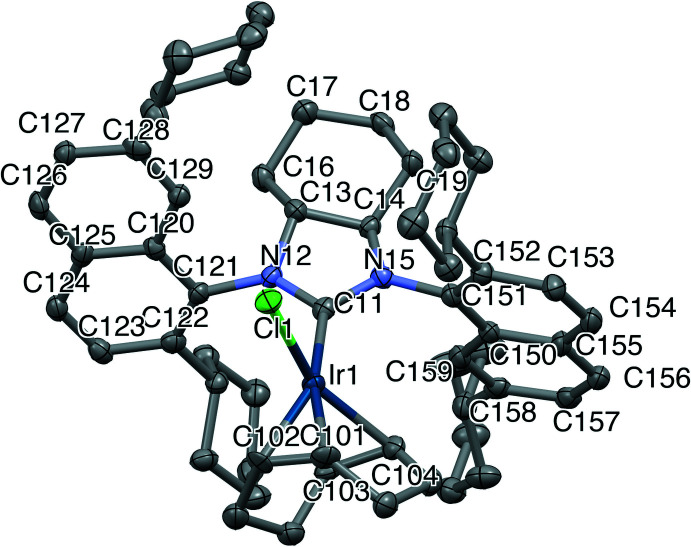
The mol­ecular structures of the title complex (II) with atom labelling. Labelling of selected aliphatic carbons has been omitted for clarity. Ellipsoids are drawn at the 50% probability level. H atoms have been omitted for clarity.

**Table 1:** Hydrogen-bond geometry (Å, °) for (I)

*D*—H⋯*A*	*D*—H	H⋯*A*	*D*⋯*A*	*D*—H⋯*A*
C17—H17*A*⋯Cl1^i^	0.99	2.79	3.773 (6)	171
C353—H353⋯Cl1	0.95	2.80	3.650 (6)	149
C372—H37*E*⋯Cl2	0.99	2.56	3.422 (6)	146

Symmetry code: (i) 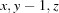.

**Table 2:** Experimental details

	(I)	(II)
Crystal data		
Chemical formula	[Ir(C_51_H_64_N_2_)Cl(C_8_H_12_)]	[Ir(C_51_H_64_N_2_)Cl(C_8_H_12_)]
*M*_r_	1040.86	1040.86
Crystal system, space group	Monoclinic, *P*2_1_	Orthorhombic, *P*2_1_2_1_2_1_
Temperature (K)	102	100
*a*, *b*, *c* (Å)	19.0004 (2), 10.4520 (1), 28.7168 (4)	16.1663 (4), 17.2811 (4), 17.6110 (3)
α, β, γ (°)	90, 107.513 (1), 90	90, 90, 90
*V* (Å^3^)	5438.59 (11)	4920.01 (19)
*Z*	4	4
Radiation type	Mo *K*α	Cu *K*α
μ (mm^−1^)	2.54	6.04
Crystal size (mm)	0.41 × 0.20 × 0.10	0.25 × 0.03 × 0.02

Data collection		
Diffractometer	Oxford Diffraction Xcalibur, Ruby, Gemini ultra	Oxford Diffraction Gemini
Absorption correction	Multi-scan *CrysAlis PRO* (Rigaku OD, 2019 ▸)	Analytical *CrysAlis PRO* (Rigaku OD, 2019 ▸)
*T*_min_, *T*_max_	0.808, 1.000	0.50, 0.90
No. of measured, independent and observed [*I* > 2σ(*I*)] reflections	349557, 38134, 32830	26296, 8782, 8164
*R*_int_	0.069	0.056
(sin θ/λ)_max_ (Å^−1^)	0.762	0.600

Refinement		
*R*[*F* ^2^ > 2σ(*F* ^2^)], *wR*(*F* ^2^), *S*	0.040, 0.083, 1.04	0.039, 0.098, 1.04
No. of reflections	38134	8782
No. of parameters	1026	568
H-atom treatment	H-atom parameters constrained	H-atom parameters constrained
Δρ_max_, Δρ_min_ (e Å^−3^)	1.57, −0.90	2.41, −0.91
Absolute structure	Flack *x* determined using 13600 quotients [(*I* ^+^)−(*I* ^−^)]/[(*I* ^+^)+(*I* ^−^)] (Parsons *et al.*, 2013 ▸)	Flack *x* determined using 3426 quotients [(*I* ^+^)−(*I* ^−^)]/[(*I* ^+^)+(*I* ^−^)] (Parsons *et al.*, 2013 ▸)
Absolute structure parameter	0.074 (4)	−0.046 (7)

Computer programs: *CrysAlis PRO* (Rigaku OD, 2019 ▸), *OLEX2.solve* (Bourhis *et al.*, 2015 ▸), *SHELXT* (Sheldrick, 2015*a* ▸), *SHELXL2014* and *SHELXL2018/3* (Sheldrick, 2015*b* ▸), *DIAMOND* (Brandenburg, 1999 ▸), *OLEX2* (Dolomanov *et al.*, 2009 ▸) and *WinGX* (Farrugia, 2012 ▸).

## supplementary crystallographic information

### [µ2-(Ra,Sa,3aR,7aR)-1,3-bis(2,7-Dicyclohexylnaphthalen-1-yl)octahydro-1H-benzo[d]imidazolidin-2-ylidene]chlorido(η4-1,5-cyclooctadiene)iridium dichloromethane monosolvate (I) Crystal data

**Table:**

[Ir(C_51_H_64_N_2_)Cl(C_8_H_12_)]	*F*(000) = 2152
*M**_r_* = 1040.86	*D*_x_ = 1.271 Mg m^−^^3^
Monoclinic, *P*2_1_	Mo *K*α radiation, λ = 0.71073 Å
*a* = 19.0004 (2) Å	Cell parameters from 82075 reflections
*b* = 10.4520 (1) Å	θ = 3.3–31.6°
*c* = 28.7168 (4) Å	µ = 2.54 mm^−^^1^
β = 107.513 (1)°	*T* = 102 K
*V* = 5438.59 (11) Å^3^	Block, clear light yellow
*Z* = 4	0.41 × 0.20 × 0.10 mm

### [µ2-(Ra,Sa,3aR,7aR)-1,3-bis(2,7-Dicyclohexylnaphthalen-1-yl)octahydro-1H-benzo[d]imidazolidin-2-ylidene]chlorido(η4-1,5-cyclooctadiene)iridium dichloromethane monosolvate (I) Data collection

**Table:**

Oxford Diffraction Xcalibur, Ruby, Gemini ultra diffractometer	38134 independent reflections
Radiation source: fine-focus sealed X-ray tube, Enhance (Mo) X-ray Source	32830 reflections with *I* > 2σ(*I*)
Graphite monochromator	*R*_int_ = 0.069
Detector resolution: 10.4738 pixels mm^-1^	θ_max_ = 32.8°, θ_min_ = 3.2°
ω scans	*h* = −28→27
Absorption correction: multi-scan CrysAlisPro (Rigaku OD, 2019)	*k* = −15→15
*T*_min_ = 0.808, *T*_max_ = 1.000	*l* = −43→43
349557 measured reflections	

### [µ2-(Ra,Sa,3aR,7aR)-1,3-bis(2,7-Dicyclohexylnaphthalen-1-yl)octahydro-1H-benzo[d]imidazolidin-2-ylidene]chlorido(η4-1,5-cyclooctadiene)iridium dichloromethane monosolvate (I) Refinement

**Table:**

Refinement on *F*^2^	Hydrogen site location: inferred from neighbouring sites
Least-squares matrix: full	H-atom parameters constrained
*R*[*F*^2^ > 2σ(*F*^2^)] = 0.040	*w* = 1/[σ^2^(*F*_o_^2^) + (0.0273*P*)^2^ + 10.0086*P*] where *P* = (*F*_o_^2^ + 2*F*_c_^2^)/3
*wR*(*F*^2^) = 0.083	(Δ/σ)_max_ = 0.003
*S* = 1.04	Δρ_max_ = 1.57 e Å^−^^3^
38134 reflections	Δρ_min_ = −0.90 e Å^−^^3^
1026 parameters	Absolute structure: Flack *x* determined using 13600 quotients [(*I*^+^)-(*I*^-^)]/[(*I*^+^)+(*I*^-^)] (Parsons *et al.*, 2013)
0 restraints	Absolute structure parameter: 0.074 (4)
Primary atom site location: iterative	

### [µ2-(Ra,Sa,3aR,7aR)-1,3-bis(2,7-Dicyclohexylnaphthalen-1-yl)octahydro-1H-benzo[d]imidazolidin-2-ylidene]chlorido(η4-1,5-cyclooctadiene)iridium dichloromethane monosolvate (I) Special details

**Table:**

Geometry. All esds (except the esd in the dihedral angle between two l.s. planes) are estimated using the full covariance matrix. The cell esds are taken into account individually in the estimation of esds in distances, angles and torsion angles; correlations between esds in cell parameters are only used when they are defined by crystal symmetry. An approximate (isotropic) treatment of cell esds is used for estimating esds involving l.s. planes.
Refinement. A solvent mask was calculated and 151 electrons were found in a volume of 927Å^3^ in 6 voids per unit cell. This is consistent with the presence of two 0.5[CH2Cl2]occupied DCM molecules per Formula Unit which account for 168 electrons per unit cell.

### [µ2-(Ra,Sa,3aR,7aR)-1,3-bis(2,7-Dicyclohexylnaphthalen-1-yl)octahydro-1H-benzo[d]imidazolidin-2-ylidene]chlorido(η4-1,5-cyclooctadiene)iridium dichloromethane monosolvate (I) Fractional atomic coordinates and isotropic or equivalent isotropic displacement parameters (Å^2^)

**Table:**

	*x*	*y*	*z*	*U*_iso_*/*U*_eq_	
Ir2	0.24765 (2)	0.56306 (2)	0.18270 (2)	0.01703 (5)	
Cl2	0.34192 (8)	0.47528 (14)	0.24718 (5)	0.0213 (3)	
N212	0.2086 (2)	0.7846 (5)	0.24086 (18)	0.0157 (9)	
N215	0.3131 (2)	0.8131 (5)	0.22408 (18)	0.0171 (9)	
C211	0.2562 (3)	0.7306 (6)	0.21910 (19)	0.0148 (10)	
C213	0.2441 (3)	0.8964 (5)	0.2698 (2)	0.0174 (9)	
H213	0.276027	0.864194	0.302075	0.021*	
C214	0.2935 (3)	0.9375 (7)	0.2411 (2)	0.0197 (10)	
H214	0.262407	0.984438	0.211772	0.024*	
C216	0.1974 (3)	1.0048 (6)	0.2798 (2)	0.0237 (12)	
H21A	0.165361	0.973654	0.298941	0.028*	
H21B	0.165754	1.041541	0.248734	0.028*	
C217	0.2519 (3)	1.1053 (5)	0.3088 (2)	0.0245 (12)	
H21C	0.277873	1.070509	0.341499	0.029*	
H21D	0.224096	1.182019	0.313317	0.029*	
C218	0.3092 (3)	1.1449 (6)	0.2835 (2)	0.0213 (12)	
H21E	0.283755	1.191601	0.253062	0.026*	
H21F	0.344964	1.204336	0.305086	0.026*	
C219	0.3513 (3)	1.0317 (5)	0.2709 (2)	0.0213 (10)	
H21G	0.383503	1.061116	0.251582	0.026*	
H21H	0.382432	0.990358	0.301072	0.026*	
C301	0.2809 (3)	0.4337 (7)	0.1343 (2)	0.0326 (5)	
H301	0.331788	0.398933	0.148996	0.039*	
C302	0.2287 (4)	0.3690 (7)	0.1491 (2)	0.0326 (5)	
H302	0.248050	0.296566	0.172121	0.039*	
C303	0.1382 (4)	0.5878 (7)	0.1378 (2)	0.0326 (5)	
H303	0.109562	0.650836	0.151056	0.039*	
C304	0.1855 (4)	0.6441 (8)	0.1157 (3)	0.0326 (5)	
H304	0.184228	0.739747	0.115761	0.039*	
C305	0.2073 (4)	0.5859 (7)	0.0722 (2)	0.0326 (5)	
H30A	0.164564	0.537322	0.051454	0.039*	
H30B	0.217229	0.656811	0.052220	0.039*	
C306	0.2714 (4)	0.5013 (6)	0.0855 (2)	0.0326 (5)	
H30C	0.316295	0.551869	0.087611	0.039*	
H30D	0.265900	0.436224	0.059581	0.039*	
C307	0.1493 (3)	0.3533 (6)	0.1165 (2)	0.0326 (5)	
H30E	0.129584	0.271135	0.124365	0.039*	
H30F	0.148994	0.349415	0.081972	0.039*	
C308	0.0981 (4)	0.4625 (8)	0.1222 (3)	0.0326 (5)	
H30G	0.059173	0.475408	0.090699	0.039*	
H30H	0.073541	0.437128	0.146714	0.039*	
C320	0.0765 (3)	0.7522 (6)	0.2270 (2)	0.0185 (11)	
C321	0.1508 (3)	0.7195 (6)	0.2540 (2)	0.0171 (11)	
C322	0.1659 (3)	0.6387 (6)	0.2938 (2)	0.0182 (11)	
C323	0.1056 (3)	0.5879 (6)	0.3077 (2)	0.0218 (11)	
H323	0.115414	0.533010	0.335226	0.026*	
C324	0.0339 (4)	0.6161 (6)	0.2823 (3)	0.0257 (13)	
H324	−0.005102	0.580103	0.292345	0.031*	
C325	0.0175 (3)	0.6978 (6)	0.2416 (2)	0.0212 (12)	
C326	−0.0562 (3)	0.7260 (6)	0.2137 (3)	0.0252 (13)	
H326	−0.095944	0.690231	0.223021	0.030*	
C327	−0.0704 (3)	0.8017 (6)	0.1748 (3)	0.0252 (13)	
H327	−0.120250	0.818701	0.156767	0.030*	
C328	−0.0130 (3)	0.8581 (6)	0.1594 (2)	0.0214 (12)	
C329	0.0584 (3)	0.8335 (6)	0.1857 (2)	0.0221 (12)	
H329	0.097067	0.872056	0.176020	0.026*	
C331	−0.0335 (3)	0.9401 (8)	0.1143 (2)	0.0276 (12)	
H331	−0.087982	0.931782	0.098749	0.033*	
C332	−0.0168 (6)	1.0809 (11)	0.1260 (4)	0.0668 (14)	
H33A	0.036657	1.092562	0.142358	0.080*	
H33B	−0.044019	1.111534	0.148309	0.080*	
C333	−0.0413 (6)	1.1610 (12)	0.0762 (4)	0.0668 (14)	
H33C	−0.095792	1.159754	0.062872	0.080*	
H33D	−0.025659	1.251165	0.083220	0.080*	
C334	−0.0095 (6)	1.1103 (11)	0.0396 (4)	0.0668 (14)	
H33E	−0.029482	1.158349	0.008755	0.080*	
H33F	0.044661	1.122848	0.050962	0.080*	
C335	−0.0256 (6)	0.9748 (11)	0.0306 (4)	0.0668 (14)	
H33G	−0.003291	0.944026	0.005620	0.080*	
H33H	−0.079701	0.962507	0.017657	0.080*	
C336	0.0043 (6)	0.8972 (11)	0.0769 (4)	0.0668 (14)	
H33I	0.058296	0.910038	0.090309	0.080*	
H33J	−0.005086	0.805091	0.069702	0.080*	
C337	0.2434 (3)	0.6096 (6)	0.3265 (2)	0.0194 (12)	
H337	0.279248	0.640793	0.309745	0.023*	
C338	0.2581 (3)	0.6820 (7)	0.3750 (2)	0.0243 (13)	
H33K	0.251686	0.774933	0.368387	0.029*	
H33L	0.221895	0.654875	0.391601	0.029*	
C339	0.3360 (4)	0.6565 (7)	0.4083 (3)	0.0291 (15)	
H33M	0.371970	0.693501	0.393222	0.035*	
H33N	0.342634	0.700327	0.439857	0.035*	
C340	0.3523 (4)	0.5174 (8)	0.4175 (3)	0.0336 (17)	
H34A	0.321712	0.482930	0.437101	0.040*	
H34B	0.404778	0.506554	0.436659	0.040*	
C341	0.3365 (3)	0.4414 (9)	0.3696 (2)	0.0261 (11)	
H34C	0.343004	0.348873	0.377063	0.031*	
H34D	0.372105	0.466728	0.352248	0.031*	
C342	0.2565 (3)	0.4666 (6)	0.3363 (2)	0.0223 (13)	
H34E	0.248431	0.420790	0.304934	0.027*	
H34F	0.220830	0.433152	0.352264	0.027*	
C350	0.3637 (3)	0.8268 (6)	0.1541 (2)	0.0176 (11)	
C351	0.3742 (3)	0.7975 (5)	0.2046 (2)	0.0150 (10)	
C352	0.4427 (3)	0.7559 (6)	0.2348 (2)	0.0173 (11)	
C353	0.4986 (3)	0.7294 (6)	0.2127 (2)	0.0212 (12)	
H353	0.545076	0.699386	0.232618	0.025*	
C354	0.4880 (3)	0.7452 (6)	0.1640 (2)	0.0214 (12)	
H354	0.525825	0.721479	0.150292	0.026*	
C355	0.4218 (3)	0.7964 (7)	0.1339 (2)	0.0212 (12)	
C356	0.4110 (4)	0.8240 (8)	0.0834 (2)	0.0275 (14)	
H356	0.447671	0.799724	0.068817	0.033*	
C357	0.3493 (4)	0.8843 (7)	0.0563 (2)	0.0306 (15)	
H357	0.344795	0.905294	0.023313	0.037*	
C358	0.2913 (3)	0.9169 (7)	0.0754 (2)	0.0240 (14)	
C359	0.2997 (3)	0.8877 (6)	0.1238 (2)	0.0201 (11)	
H359	0.261152	0.909279	0.137134	0.024*	
C361	0.2239 (4)	0.9894 (8)	0.0436 (3)	0.0315 (16)	
H361	0.186886	0.995606	0.061959	0.038*	
C362	0.2448 (4)	1.1234 (8)	0.0332 (3)	0.0377 (17)	
H36A	0.285622	1.118723	0.018411	0.045*	
H36B	0.263095	1.170796	0.064346	0.045*	
C363	0.1799 (6)	1.1978 (9)	−0.0015 (3)	0.047 (2)	
H36C	0.196748	1.283784	−0.007909	0.056*	
H36D	0.140237	1.208607	0.013994	0.056*	
C364	0.1497 (5)	1.1238 (9)	−0.0502 (3)	0.045 (2)	
H36E	0.107051	1.170183	−0.072058	0.054*	
H36F	0.188442	1.117692	−0.066712	0.054*	
C365	0.1258 (4)	0.9889 (8)	−0.0399 (3)	0.0357 (16)	
H36G	0.083824	0.995367	−0.026343	0.043*	
H36H	0.109041	0.940228	−0.070884	0.043*	
C366	0.1880 (4)	0.9188 (9)	−0.0046 (3)	0.0348 (18)	
H36I	0.226388	0.901344	−0.020652	0.042*	
H36J	0.169318	0.835322	0.002894	0.042*	
C367	0.4631 (3)	0.7462 (6)	0.2898 (2)	0.0185 (11)	
H367	0.416957	0.752250	0.299600	0.022*	
C368	0.5148 (3)	0.8574 (5)	0.3138 (2)	0.0211 (11)	
H36K	0.561480	0.850079	0.305386	0.025*	
H36L	0.491312	0.939632	0.300637	0.025*	
C369	0.5319 (3)	0.8575 (6)	0.3689 (2)	0.0273 (12)	
H36M	0.564908	0.930145	0.382945	0.033*	
H36N	0.485669	0.868168	0.377628	0.033*	
C370	0.5687 (4)	0.7335 (7)	0.3898 (2)	0.0282 (13)	
H37A	0.576180	0.732214	0.425389	0.034*	
H37B	0.617801	0.728805	0.384539	0.034*	
C371	0.5236 (4)	0.6172 (7)	0.3666 (2)	0.0272 (14)	
H37C	0.477787	0.614127	0.376275	0.033*	
H37D	0.552231	0.538570	0.378936	0.033*	
C372	0.5036 (3)	0.6210 (6)	0.3107 (2)	0.0222 (12)	
H37E	0.471582	0.547056	0.296726	0.027*	
H37F	0.549141	0.613733	0.300914	0.027*	
Ir1	0.75763 (2)	0.442289 (6)	0.31455 (2)	0.01599 (5)	
Cl1	0.65873 (8)	0.51820 (14)	0.25058 (5)	0.0196 (3)	
N12	0.6958 (2)	0.1776 (4)	0.28132 (17)	0.0137 (8)	
N15	0.7916 (2)	0.2176 (5)	0.25652 (18)	0.0160 (9)	
C11	0.7485 (3)	0.2672 (5)	0.2814 (2)	0.0152 (10)	
C13	0.6977 (3)	0.0714 (6)	0.24819 (19)	0.0165 (9)	
H13	0.666707	0.096789	0.214734	0.020*	
C14	0.7777 (3)	0.0774 (5)	0.24884 (19)	0.0178 (9)	
H14	0.808040	0.032034	0.278832	0.021*	
C16	0.6759 (3)	−0.0611 (5)	0.25724 (18)	0.0194 (9)	
H16A	0.623923	−0.063286	0.257438	0.023*	
H16B	0.707814	−0.093463	0.288972	0.023*	
C17	0.6861 (3)	−0.1428 (6)	0.2143 (2)	0.0200 (12)	
H17A	0.676466	−0.233818	0.219790	0.024*	
H17B	0.649442	−0.115434	0.183455	0.024*	
C18	0.7640 (3)	−0.1302 (5)	0.2091 (2)	0.0232 (12)	
H18A	0.765699	−0.177227	0.179535	0.028*	
H18B	0.799635	−0.171321	0.237626	0.028*	
C19	0.7884 (3)	0.0101 (6)	0.2054 (2)	0.0239 (13)	
H19A	0.840850	0.013483	0.206178	0.029*	
H19B	0.757875	0.050061	0.174721	0.029*	
C101	0.7843 (3)	0.6404 (6)	0.3353 (2)	0.0279 (4)	
H101	0.757692	0.702551	0.309509	0.033*	
C102	0.7402 (3)	0.5923 (5)	0.3623 (2)	0.0279 (4)	
H102	0.688958	0.627573	0.353075	0.033*	
C103	0.8192 (4)	0.3609 (7)	0.3818 (2)	0.0279 (4)	
H103	0.821375	0.265374	0.381298	0.033*	
C104	0.8667 (4)	0.4199 (6)	0.3602 (2)	0.0279 (4)	
H104	0.896129	0.357933	0.347071	0.033*	
C105	0.9076 (3)	0.5452 (7)	0.3779 (2)	0.0279 (4)	
H10A	0.915995	0.553441	0.413484	0.033*	
H10B	0.956511	0.542038	0.372246	0.033*	
C106	0.8665 (3)	0.6616 (5)	0.3527 (2)	0.0279 (4)	
H10C	0.877760	0.734934	0.375571	0.033*	
H10D	0.883792	0.683545	0.324398	0.033*	
C107	0.7722 (3)	0.5569 (6)	0.4158 (2)	0.0279 (4)	
H10E	0.734874	0.574246	0.432690	0.033*	
H10F	0.815462	0.612149	0.430722	0.033*	
C108	0.7964 (4)	0.4145 (6)	0.4240 (2)	0.0279 (4)	
H10G	0.838336	0.407369	0.454338	0.033*	
H10H	0.755082	0.363125	0.428341	0.033*	
C120	0.6444 (3)	0.1727 (6)	0.3502 (2)	0.0166 (10)	
C121	0.6343 (3)	0.2016 (6)	0.2999 (2)	0.0158 (10)	
C122	0.5662 (3)	0.2416 (5)	0.2694 (2)	0.0164 (10)	
C123	0.5090 (3)	0.2659 (6)	0.2904 (2)	0.0195 (11)	
H123	0.461811	0.291032	0.269861	0.023*	
C124	0.5198 (3)	0.2541 (6)	0.3394 (2)	0.0213 (12)	
H124	0.481766	0.278862	0.352735	0.026*	
C125	0.5871 (3)	0.2053 (6)	0.3706 (2)	0.0181 (11)	
C126	0.5963 (4)	0.1806 (7)	0.4203 (2)	0.0251 (13)	
H126	0.558198	0.203617	0.433844	0.030*	
C127	0.6585 (3)	0.1246 (6)	0.4493 (2)	0.0231 (12)	
H127	0.664435	0.112452	0.483034	0.028*	
C128	0.7152 (3)	0.0840 (6)	0.4294 (2)	0.0204 (12)	
C129	0.7081 (3)	0.1103 (6)	0.3814 (2)	0.0184 (11)	
H129	0.746641	0.086152	0.368446	0.022*	
C131	0.7810 (3)	0.0144 (6)	0.4618 (2)	0.0202 (12)	
H131	0.818482	0.007301	0.443812	0.024*	
C132	0.8168 (4)	0.0867 (7)	0.5096 (2)	0.0253 (14)	
H13A	0.778291	0.108987	0.525029	0.030*	
H13B	0.838418	0.167509	0.502192	0.030*	
C133	0.8781 (4)	0.0075 (8)	0.5463 (3)	0.0317 (15)	
H13C	0.920313	−0.003124	0.533209	0.038*	
H13D	0.895755	0.054703	0.577571	0.038*	
C134	0.8511 (4)	−0.1199 (7)	0.5554 (3)	0.0303 (15)	
H13E	0.811455	−0.109400	0.570918	0.036*	
H13F	0.891970	−0.167986	0.578314	0.036*	
C135	0.8220 (4)	−0.1948 (8)	0.5090 (3)	0.0309 (16)	
H13G	0.804220	−0.279133	0.516311	0.037*	
H13H	0.862370	−0.209503	0.494357	0.037*	
C136	0.7595 (4)	−0.1240 (7)	0.4728 (2)	0.0249 (13)	
H13I	0.716941	−0.119382	0.485869	0.030*	
H13J	0.743763	−0.173030	0.441867	0.030*	
C137	0.5481 (3)	0.2554 (6)	0.2145 (2)	0.0181 (10)	
H137	0.595409	0.268188	0.206612	0.022*	
C138	0.4966 (3)	0.3689 (6)	0.1929 (2)	0.0217 (12)	
H13K	0.448862	0.356975	0.199691	0.026*	
H13L	0.519068	0.449219	0.208873	0.026*	
C139	0.4831 (4)	0.3792 (7)	0.1384 (3)	0.0271 (13)	
H13M	0.530398	0.397925	0.131948	0.033*	
H13N	0.449066	0.451340	0.125555	0.033*	
C140	0.4501 (4)	0.2559 (7)	0.1116 (3)	0.0343 (15)	
H14A	0.399638	0.243385	0.114057	0.041*	
H14B	0.446470	0.263555	0.076614	0.041*	
C141	0.4977 (4)	0.1405 (6)	0.1335 (2)	0.0300 (13)	
H14C	0.545604	0.147017	0.126618	0.036*	
H14D	0.472884	0.061398	0.117918	0.036*	
C142	0.5115 (3)	0.1321 (5)	0.1889 (2)	0.0231 (11)	
H14E	0.543882	0.058023	0.202082	0.028*	
H14F	0.464054	0.118437	0.195802	0.028*	
C150	0.9256 (3)	0.2418 (6)	0.2704 (2)	0.0157 (11)	
C151	0.8521 (3)	0.2766 (6)	0.2433 (2)	0.0163 (11)	
C152	0.8374 (3)	0.3537 (6)	0.2028 (2)	0.0177 (11)	
C153	0.8989 (3)	0.3978 (6)	0.1882 (2)	0.0225 (12)	
H153	0.889622	0.451170	0.160189	0.027*	
C154	0.9695 (3)	0.3671 (6)	0.2124 (2)	0.0219 (12)	
H154	1.008483	0.398263	0.201170	0.026*	
C155	0.9853 (3)	0.2884 (6)	0.2544 (2)	0.0194 (11)	
C156	1.0585 (3)	0.2584 (6)	0.2821 (2)	0.0219 (12)	
H156	1.098346	0.288936	0.271654	0.026*	
C157	1.0729 (3)	0.1853 (6)	0.3241 (2)	0.0201 (11)	
H157	1.122563	0.165820	0.341997	0.024*	
C158	1.0152 (3)	0.1396 (6)	0.3407 (2)	0.0177 (11)	
C159	0.9426 (3)	0.1659 (6)	0.3134 (2)	0.0146 (10)	
H159	0.903411	0.132154	0.323795	0.018*	
C161	1.0328 (3)	0.0626 (8)	0.3876 (2)	0.0219 (11)	
H161	1.087167	0.070403	0.403622	0.026*	
C162	0.9954 (4)	0.1150 (6)	0.4237 (2)	0.0249 (11)	
H16C	0.941226	0.106265	0.409687	0.030*	
H16D	1.006904	0.207243	0.429120	0.030*	
C163	1.0206 (4)	0.0449 (8)	0.4727 (2)	0.0358 (15)	
H16E	1.074072	0.058787	0.488154	0.043*	
H16F	0.993940	0.079033	0.494781	0.043*	
C164	1.0049 (4)	−0.0978 (8)	0.4644 (3)	0.0399 (17)	
H16G	0.950892	−0.111594	0.452852	0.048*	
H16H	1.024759	−0.143590	0.495820	0.048*	
C165	1.0394 (4)	−0.1538 (7)	0.4270 (4)	0.051 (2)	
H16I	1.023792	−0.244145	0.420551	0.061*	
H16J	1.093797	−0.152271	0.440771	0.061*	
C166	1.0165 (4)	−0.0789 (6)	0.3789 (3)	0.0310 (13)	
H16K	1.043491	−0.112587	0.356936	0.037*	
H16L	0.963020	−0.091047	0.362649	0.037*	
C167	0.7610 (3)	0.3886 (6)	0.1711 (2)	0.0180 (11)	
H167	0.724884	0.357210	0.187564	0.022*	
C168	0.7433 (4)	0.3232 (7)	0.1212 (2)	0.0250 (13)	
H16M	0.779700	0.350791	0.104780	0.030*	
H16N	0.748168	0.229443	0.126012	0.030*	
C169	0.6663 (4)	0.3534 (8)	0.0883 (3)	0.0324 (16)	
H16O	0.629369	0.316970	0.102692	0.039*	
H16P	0.658892	0.313254	0.055992	0.039*	
C170	0.6546 (4)	0.4982 (7)	0.0821 (3)	0.0287 (14)	
H17C	0.686052	0.532260	0.062991	0.034*	
H17D	0.602499	0.515576	0.063474	0.034*	
C171	0.6735 (3)	0.5670 (8)	0.1313 (2)	0.0248 (11)	
H17E	0.669617	0.660517	0.125714	0.030*	
H17F	0.637146	0.542439	0.148241	0.030*	
C172	0.7499 (3)	0.5351 (6)	0.1634 (2)	0.0236 (14)	
H17G	0.758504	0.577230	0.195502	0.028*	
H17H	0.786598	0.568840	0.148317	0.028*	

### [µ2-(Ra,Sa,3aR,7aR)-1,3-bis(2,7-Dicyclohexylnaphthalen-1-yl)octahydro-1H-benzo[d]imidazolidin-2-ylidene]chlorido(η4-1,5-cyclooctadiene)iridium dichloromethane monosolvate (I) Atomic displacement parameters (Å^2^)

**Table:**

	*U*^11^	*U*^22^	*U*^33^	*U*^12^	*U*^13^	*U*^23^
Ir2	0.01774 (9)	0.01498 (12)	0.01705 (10)	−0.00075 (8)	0.00323 (8)	−0.00294 (9)
Cl2	0.0221 (6)	0.0206 (8)	0.0192 (6)	0.0038 (5)	0.0034 (5)	0.0006 (5)
N212	0.016 (2)	0.012 (2)	0.020 (2)	0.0001 (17)	0.0070 (18)	−0.0019 (18)
N215	0.0160 (19)	0.013 (2)	0.025 (2)	0.0010 (16)	0.0098 (18)	−0.0027 (17)
C211	0.014 (2)	0.020 (3)	0.009 (2)	0.0009 (19)	0.0018 (18)	0.002 (2)
C213	0.017 (2)	0.014 (2)	0.024 (2)	−0.0019 (17)	0.0103 (19)	−0.0039 (18)
C214	0.018 (2)	0.015 (2)	0.028 (3)	−0.002 (2)	0.0114 (19)	−0.004 (2)
C216	0.020 (2)	0.017 (3)	0.039 (3)	−0.003 (2)	0.016 (2)	−0.008 (2)
C217	0.026 (3)	0.014 (2)	0.039 (3)	−0.0016 (19)	0.019 (3)	−0.008 (2)
C218	0.022 (3)	0.016 (3)	0.027 (3)	−0.004 (2)	0.009 (2)	−0.007 (2)
C219	0.020 (2)	0.016 (2)	0.030 (3)	−0.0033 (17)	0.011 (2)	−0.0051 (19)
C301	0.0349 (11)	0.0321 (12)	0.0269 (10)	0.0001 (10)	0.0032 (9)	−0.0072 (9)
C302	0.0349 (11)	0.0321 (12)	0.0269 (10)	0.0001 (10)	0.0032 (9)	−0.0072 (9)
C303	0.0349 (11)	0.0321 (12)	0.0269 (10)	0.0001 (10)	0.0032 (9)	−0.0072 (9)
C304	0.0349 (11)	0.0321 (12)	0.0269 (10)	0.0001 (10)	0.0032 (9)	−0.0072 (9)
C305	0.0349 (11)	0.0321 (12)	0.0269 (10)	0.0001 (10)	0.0032 (9)	−0.0072 (9)
C306	0.0349 (11)	0.0321 (12)	0.0269 (10)	0.0001 (10)	0.0032 (9)	−0.0072 (9)
C307	0.0349 (11)	0.0321 (12)	0.0269 (10)	0.0001 (10)	0.0032 (9)	−0.0072 (9)
C308	0.0349 (11)	0.0321 (12)	0.0269 (10)	0.0001 (10)	0.0032 (9)	−0.0072 (9)
C320	0.019 (2)	0.017 (3)	0.021 (3)	0.002 (2)	0.009 (2)	−0.003 (2)
C321	0.016 (2)	0.016 (3)	0.022 (3)	−0.0013 (19)	0.009 (2)	−0.003 (2)
C322	0.020 (2)	0.015 (3)	0.022 (3)	0.000 (2)	0.010 (2)	−0.001 (2)
C323	0.024 (2)	0.017 (3)	0.026 (3)	0.000 (2)	0.009 (2)	0.001 (2)
C324	0.026 (3)	0.023 (3)	0.036 (3)	−0.007 (2)	0.021 (3)	−0.004 (2)
C325	0.017 (2)	0.022 (3)	0.029 (3)	−0.001 (2)	0.013 (2)	−0.006 (2)
C326	0.016 (2)	0.025 (3)	0.038 (3)	0.000 (2)	0.012 (2)	−0.002 (3)
C327	0.016 (2)	0.025 (3)	0.036 (3)	−0.001 (2)	0.008 (2)	−0.010 (3)
C328	0.019 (3)	0.018 (3)	0.027 (3)	0.001 (2)	0.006 (2)	−0.003 (2)
C329	0.021 (3)	0.016 (3)	0.033 (3)	0.000 (2)	0.014 (3)	−0.002 (3)
C331	0.017 (2)	0.025 (3)	0.037 (3)	−0.004 (3)	0.003 (2)	0.001 (4)
C332	0.061 (3)	0.068 (3)	0.061 (3)	−0.010 (2)	0.003 (2)	0.024 (2)
C333	0.061 (3)	0.068 (3)	0.061 (3)	−0.010 (2)	0.003 (2)	0.024 (2)
C334	0.061 (3)	0.068 (3)	0.061 (3)	−0.010 (2)	0.003 (2)	0.024 (2)
C335	0.061 (3)	0.068 (3)	0.061 (3)	−0.010 (2)	0.003 (2)	0.024 (2)
C336	0.061 (3)	0.068 (3)	0.061 (3)	−0.010 (2)	0.003 (2)	0.024 (2)
C337	0.023 (3)	0.018 (3)	0.020 (3)	−0.002 (2)	0.010 (2)	−0.001 (2)
C338	0.026 (3)	0.028 (3)	0.022 (3)	0.000 (2)	0.010 (2)	0.002 (3)
C339	0.028 (3)	0.035 (4)	0.025 (3)	−0.011 (3)	0.009 (3)	−0.006 (3)
C340	0.028 (3)	0.050 (5)	0.022 (3)	−0.001 (3)	0.006 (3)	0.002 (3)
C341	0.026 (3)	0.028 (3)	0.023 (3)	−0.001 (3)	0.007 (2)	−0.002 (3)
C342	0.027 (3)	0.020 (4)	0.022 (3)	−0.006 (2)	0.010 (2)	−0.002 (2)
C350	0.020 (2)	0.015 (3)	0.020 (2)	−0.004 (2)	0.008 (2)	−0.002 (2)
C351	0.015 (2)	0.012 (3)	0.019 (2)	−0.0049 (18)	0.0077 (19)	−0.0050 (19)
C352	0.016 (2)	0.013 (3)	0.025 (3)	0.0007 (18)	0.010 (2)	−0.002 (2)
C353	0.019 (2)	0.019 (3)	0.030 (3)	0.004 (2)	0.013 (2)	0.003 (2)
C354	0.018 (2)	0.022 (3)	0.028 (3)	−0.001 (2)	0.012 (2)	−0.008 (2)
C355	0.021 (3)	0.026 (3)	0.019 (3)	−0.007 (2)	0.011 (2)	−0.007 (2)
C356	0.023 (3)	0.041 (4)	0.023 (3)	−0.001 (3)	0.013 (2)	−0.004 (3)
C357	0.032 (3)	0.044 (4)	0.019 (3)	−0.011 (3)	0.012 (2)	−0.005 (3)
C358	0.024 (3)	0.030 (4)	0.019 (3)	−0.005 (2)	0.008 (2)	−0.001 (2)
C359	0.019 (2)	0.025 (3)	0.018 (2)	−0.004 (2)	0.008 (2)	−0.002 (2)
C361	0.028 (3)	0.044 (4)	0.022 (3)	−0.008 (3)	0.006 (3)	−0.004 (3)
C362	0.039 (4)	0.034 (4)	0.030 (3)	0.000 (3)	−0.004 (3)	−0.007 (3)
C363	0.063 (5)	0.027 (4)	0.040 (4)	0.000 (4)	0.000 (4)	−0.008 (3)
C364	0.050 (5)	0.044 (5)	0.033 (4)	0.010 (4)	0.000 (4)	0.006 (4)
C365	0.036 (4)	0.038 (4)	0.028 (3)	−0.008 (3)	0.002 (3)	−0.002 (3)
C366	0.035 (3)	0.040 (5)	0.024 (3)	−0.007 (3)	0.000 (3)	0.002 (3)
C367	0.015 (2)	0.021 (3)	0.021 (3)	−0.0001 (19)	0.008 (2)	0.000 (2)
C368	0.021 (2)	0.018 (3)	0.027 (3)	−0.001 (2)	0.011 (2)	−0.002 (2)
C369	0.028 (3)	0.027 (3)	0.027 (3)	−0.003 (2)	0.008 (2)	−0.008 (2)
C370	0.033 (3)	0.025 (3)	0.026 (3)	0.002 (2)	0.008 (3)	−0.001 (2)
C371	0.023 (3)	0.029 (4)	0.024 (3)	−0.002 (2)	0.000 (2)	0.002 (3)
C372	0.023 (3)	0.016 (3)	0.027 (3)	0.001 (2)	0.006 (2)	0.001 (2)
Ir1	0.01665 (9)	0.01127 (10)	0.01773 (10)	−0.00002 (8)	0.00169 (8)	−0.00114 (9)
Cl1	0.0182 (6)	0.0165 (7)	0.0216 (6)	0.0027 (5)	0.0024 (5)	0.0001 (5)
N12	0.0149 (18)	0.009 (2)	0.018 (2)	0.0004 (15)	0.0059 (16)	0.0004 (16)
N15	0.0120 (19)	0.015 (2)	0.021 (2)	−0.0045 (17)	0.0054 (18)	−0.0008 (19)
C11	0.014 (2)	0.009 (2)	0.021 (3)	0.0026 (18)	0.003 (2)	0.003 (2)
C13	0.0161 (19)	0.014 (2)	0.020 (2)	−0.0041 (19)	0.0071 (17)	−0.005 (2)
C14	0.0148 (19)	0.014 (2)	0.028 (2)	0.0013 (18)	0.0111 (18)	−0.002 (2)
C16	0.019 (2)	0.015 (2)	0.026 (2)	−0.0051 (19)	0.0086 (18)	−0.001 (2)
C17	0.022 (3)	0.014 (3)	0.028 (3)	−0.004 (2)	0.014 (2)	−0.004 (2)
C18	0.024 (3)	0.012 (2)	0.040 (3)	0.0004 (19)	0.019 (2)	−0.005 (2)
C19	0.026 (3)	0.020 (3)	0.032 (3)	−0.004 (2)	0.020 (3)	−0.007 (2)
C101	0.0309 (10)	0.0198 (10)	0.0278 (10)	0.0019 (8)	0.0009 (8)	−0.0049 (8)
C102	0.0309 (10)	0.0198 (10)	0.0278 (10)	0.0019 (8)	0.0009 (8)	−0.0049 (8)
C103	0.0309 (10)	0.0198 (10)	0.0278 (10)	0.0019 (8)	0.0009 (8)	−0.0049 (8)
C104	0.0309 (10)	0.0198 (10)	0.0278 (10)	0.0019 (8)	0.0009 (8)	−0.0049 (8)
C105	0.0309 (10)	0.0198 (10)	0.0278 (10)	0.0019 (8)	0.0009 (8)	−0.0049 (8)
C106	0.0309 (10)	0.0198 (10)	0.0278 (10)	0.0019 (8)	0.0009 (8)	−0.0049 (8)
C107	0.0309 (10)	0.0198 (10)	0.0278 (10)	0.0019 (8)	0.0009 (8)	−0.0049 (8)
C108	0.0309 (10)	0.0198 (10)	0.0278 (10)	0.0019 (8)	0.0009 (8)	−0.0049 (8)
C120	0.016 (2)	0.015 (3)	0.022 (3)	−0.0012 (18)	0.009 (2)	−0.002 (2)
C121	0.014 (2)	0.014 (3)	0.021 (2)	0.0025 (18)	0.0070 (19)	0.001 (2)
C122	0.018 (2)	0.011 (2)	0.020 (2)	−0.0026 (18)	0.006 (2)	0.0008 (19)
C123	0.015 (2)	0.014 (3)	0.030 (3)	0.0000 (19)	0.007 (2)	−0.003 (2)
C124	0.020 (3)	0.018 (3)	0.032 (3)	0.002 (2)	0.017 (2)	0.001 (2)
C125	0.017 (2)	0.015 (3)	0.024 (3)	0.0005 (19)	0.008 (2)	−0.001 (2)
C126	0.031 (3)	0.024 (3)	0.027 (3)	−0.001 (2)	0.018 (3)	−0.004 (3)
C127	0.023 (3)	0.028 (3)	0.020 (3)	0.001 (2)	0.008 (2)	−0.002 (2)
C128	0.022 (2)	0.019 (3)	0.020 (2)	0.000 (2)	0.006 (2)	0.001 (2)
C129	0.015 (2)	0.016 (3)	0.023 (3)	−0.0028 (18)	0.003 (2)	−0.001 (2)
C131	0.022 (3)	0.021 (3)	0.019 (3)	0.003 (2)	0.009 (2)	0.003 (2)
C132	0.032 (3)	0.018 (4)	0.023 (3)	−0.005 (2)	0.004 (2)	−0.002 (2)
C133	0.025 (3)	0.043 (4)	0.023 (3)	0.006 (3)	0.001 (2)	0.009 (3)
C134	0.032 (3)	0.032 (4)	0.028 (3)	0.007 (3)	0.011 (3)	0.005 (3)
C135	0.031 (3)	0.029 (4)	0.033 (4)	0.010 (3)	0.010 (3)	0.009 (3)
C136	0.028 (3)	0.021 (3)	0.026 (3)	−0.001 (2)	0.008 (2)	0.004 (2)
C137	0.018 (2)	0.014 (3)	0.022 (3)	0.0007 (18)	0.004 (2)	−0.001 (2)
C138	0.017 (2)	0.022 (3)	0.023 (3)	0.001 (2)	0.001 (2)	−0.002 (2)
C139	0.023 (3)	0.024 (3)	0.032 (3)	0.002 (2)	0.006 (3)	0.004 (3)
C140	0.035 (3)	0.036 (4)	0.026 (3)	−0.001 (3)	0.001 (3)	−0.003 (3)
C141	0.037 (3)	0.024 (3)	0.028 (3)	−0.005 (3)	0.008 (3)	−0.005 (2)
C142	0.022 (3)	0.014 (3)	0.031 (3)	0.000 (2)	0.004 (2)	0.001 (2)
C150	0.013 (2)	0.013 (3)	0.022 (3)	−0.0020 (18)	0.008 (2)	−0.003 (2)
C151	0.017 (2)	0.012 (3)	0.022 (3)	−0.0062 (19)	0.010 (2)	−0.005 (2)
C152	0.018 (2)	0.016 (3)	0.020 (3)	−0.0024 (19)	0.008 (2)	−0.003 (2)
C153	0.026 (3)	0.024 (3)	0.023 (3)	−0.005 (2)	0.014 (2)	0.000 (2)
C154	0.019 (2)	0.027 (3)	0.023 (3)	−0.004 (2)	0.012 (2)	0.000 (2)
C155	0.019 (2)	0.019 (3)	0.022 (3)	−0.002 (2)	0.009 (2)	−0.002 (2)
C156	0.015 (2)	0.027 (3)	0.028 (3)	−0.002 (2)	0.013 (2)	−0.004 (2)
C157	0.013 (2)	0.019 (3)	0.029 (3)	0.0035 (19)	0.009 (2)	0.001 (2)
C158	0.016 (2)	0.014 (3)	0.024 (3)	0.002 (2)	0.008 (2)	−0.003 (2)
C159	0.015 (2)	0.013 (3)	0.016 (2)	−0.0013 (19)	0.004 (2)	0.001 (2)
C161	0.013 (2)	0.023 (3)	0.027 (3)	−0.002 (3)	0.003 (2)	0.011 (3)
C162	0.029 (3)	0.024 (3)	0.019 (2)	0.005 (2)	0.004 (2)	0.002 (2)
C163	0.032 (3)	0.046 (4)	0.026 (3)	−0.008 (3)	0.003 (2)	0.009 (3)
C164	0.027 (3)	0.050 (4)	0.036 (3)	−0.013 (3)	−0.002 (3)	0.017 (3)
C165	0.033 (3)	0.011 (3)	0.091 (6)	0.005 (2)	−0.007 (4)	0.013 (3)
C166	0.036 (3)	0.009 (3)	0.043 (3)	0.004 (2)	0.005 (3)	−0.001 (2)
C167	0.017 (2)	0.017 (3)	0.019 (3)	−0.003 (2)	0.005 (2)	0.003 (2)
C168	0.031 (3)	0.021 (3)	0.023 (3)	−0.008 (2)	0.008 (3)	−0.006 (2)
C169	0.032 (3)	0.040 (5)	0.023 (3)	−0.006 (3)	0.005 (3)	−0.004 (3)
C170	0.027 (3)	0.034 (4)	0.019 (3)	0.002 (3)	−0.001 (2)	0.006 (3)
C171	0.024 (2)	0.025 (3)	0.023 (3)	0.004 (3)	0.005 (2)	0.006 (3)
C172	0.025 (3)	0.023 (4)	0.021 (3)	0.000 (2)	0.004 (2)	0.004 (2)

### [µ2-(Ra,Sa,3aR,7aR)-1,3-bis(2,7-Dicyclohexylnaphthalen-1-yl)octahydro-1H-benzo[d]imidazolidin-2-ylidene]chlorido(η4-1,5-cyclooctadiene)iridium dichloromethane monosolvate (I) Geometric parameters (Å, º)

**Table:**

Ir2—Cl2	2.3415 (15)	Ir1—Cl1	2.3361 (15)
Ir2—C211	2.021 (6)	Ir1—C11	2.046 (6)
Ir2—C301	2.165 (6)	Ir1—C101	2.172 (6)
Ir2—C302	2.228 (6)	Ir1—C102	2.173 (6)
Ir2—C303	2.106 (7)	Ir1—C103	2.112 (7)
Ir2—C304	2.109 (7)	Ir1—C104	2.106 (6)
N212—C211	1.367 (6)	N12—C11	1.371 (7)
N212—C213	1.475 (7)	N12—C13	1.469 (7)
N212—C321	1.434 (7)	N12—C121	1.447 (6)
N215—C211	1.357 (7)	N15—C11	1.342 (7)
N215—C214	1.476 (8)	N15—C14	1.494 (7)
N215—C351	1.439 (6)	N15—C151	1.452 (6)
C213—H213	1.0000	C13—H13	1.0000
C213—C214	1.487 (7)	C13—C14	1.516 (6)
C213—C216	1.519 (7)	C13—C16	1.490 (8)
C214—H214	1.0000	C14—H14	1.0000
C214—C219	1.530 (8)	C14—C19	1.498 (7)
C216—H21A	0.9900	C16—H16A	0.9900
C216—H21B	0.9900	C16—H16B	0.9900
C216—C217	1.534 (8)	C16—C17	1.560 (7)
C217—H21C	0.9900	C17—H17A	0.9900
C217—H21D	0.9900	C17—H17B	0.9900
C217—C218	1.536 (7)	C17—C18	1.537 (7)
C218—H21E	0.9900	C18—H18A	0.9900
C218—H21F	0.9900	C18—H18B	0.9900
C218—C219	1.531 (7)	C18—C19	1.552 (8)
C219—H21G	0.9900	C19—H19A	0.9900
C219—H21H	0.9900	C19—H19B	0.9900
C301—H301	1.0000	C101—H101	1.0000
C301—C302	1.369 (9)	C101—C102	1.394 (9)
C301—C306	1.530 (9)	C101—C106	1.507 (8)
C302—H302	1.0000	C102—H102	1.0000
C302—C307	1.527 (8)	C102—C107	1.518 (8)
C303—H303	1.0000	C103—H103	1.0000
C303—C304	1.379 (10)	C103—C104	1.384 (9)
C303—C308	1.514 (10)	C103—C108	1.513 (9)
C304—H304	1.0000	C104—H104	1.0000
C304—C305	1.553 (9)	C104—C105	1.530 (9)
C305—H30A	0.9900	C105—H10A	0.9900
C305—H30B	0.9900	C105—H10B	0.9900
C305—C306	1.460 (9)	C105—C106	1.508 (9)
C306—H30C	0.9900	C106—H10C	0.9900
C306—H30D	0.9900	C106—H10D	0.9900
C307—H30E	0.9900	C107—H10E	0.9900
C307—H30F	0.9900	C107—H10F	0.9900
C307—C308	1.541 (9)	C107—C108	1.554 (9)
C308—H30G	0.9900	C108—H10G	0.9900
C308—H30H	0.9900	C108—H10H	0.9900
C320—C321	1.433 (8)	C120—C121	1.430 (8)
C320—C325	1.429 (7)	C120—C125	1.423 (7)
C320—C329	1.413 (9)	C120—C129	1.428 (8)
C321—C322	1.380 (8)	C121—C122	1.390 (8)
C322—C323	1.424 (7)	C122—C123	1.416 (7)
C322—C337	1.519 (9)	C122—C137	1.516 (8)
C323—H323	0.9500	C123—H123	0.9500
C323—C324	1.370 (9)	C123—C124	1.364 (9)
C324—H324	0.9500	C124—H124	0.9500
C324—C325	1.405 (9)	C124—C125	1.416 (9)
C325—C326	1.418 (9)	C125—C126	1.410 (8)
C326—H326	0.9500	C126—H126	0.9500
C326—C327	1.330 (10)	C126—C127	1.356 (9)
C327—H327	0.9500	C127—H127	0.9500
C327—C328	1.422 (8)	C127—C128	1.428 (8)
C328—C329	1.363 (9)	C128—C129	1.372 (8)
C328—C331	1.503 (10)	C128—C131	1.502 (9)
C329—H329	0.9500	C129—H129	0.9500
C331—H331	1.0000	C131—H131	1.0000
C331—C332	1.521 (15)	C131—C132	1.537 (9)
C331—C336	1.528 (13)	C131—C136	1.562 (9)
C332—H33A	0.9900	C132—H13A	0.9900
C332—H33B	0.9900	C132—H13B	0.9900
C332—C333	1.601 (13)	C132—C133	1.553 (10)
C333—H33C	0.9900	C133—H13C	0.9900
C333—H33D	0.9900	C133—H13D	0.9900
C333—C334	1.460 (16)	C133—C134	1.478 (11)
C334—H33E	0.9900	C134—H13E	0.9900
C334—H33F	0.9900	C134—H13F	0.9900
C334—C335	1.456 (15)	C134—C135	1.501 (11)
C335—H33G	0.9900	C135—H13G	0.9900
C335—H33H	0.9900	C135—H13H	0.9900
C335—C336	1.516 (14)	C135—C136	1.515 (10)
C336—H33I	0.9900	C136—H13I	0.9900
C336—H33J	0.9900	C136—H13J	0.9900
C337—H337	1.0000	C137—H137	1.0000
C337—C338	1.535 (9)	C137—C138	1.544 (8)
C337—C342	1.527 (9)	C137—C142	1.541 (8)
C338—H33K	0.9900	C138—H13K	0.9900
C338—H33L	0.9900	C138—H13L	0.9900
C338—C339	1.525 (10)	C138—C139	1.512 (9)
C339—H33M	0.9900	C139—H13M	0.9900
C339—H33N	0.9900	C139—H13N	0.9900
C339—C340	1.493 (11)	C139—C140	1.536 (10)
C340—H34A	0.9900	C140—H14A	0.9900
C340—H34B	0.9900	C140—H14B	0.9900
C340—C341	1.538 (10)	C140—C141	1.525 (10)
C341—H34C	0.9900	C141—H14C	0.9900
C341—H34D	0.9900	C141—H14D	0.9900
C341—C342	1.555 (9)	C141—C142	1.536 (9)
C342—H34E	0.9900	C142—H14E	0.9900
C342—H34F	0.9900	C142—H14F	0.9900
C350—C351	1.437 (8)	C150—C151	1.427 (8)
C350—C355	1.429 (7)	C150—C155	1.431 (7)
C350—C359	1.415 (9)	C150—C159	1.419 (8)
C351—C352	1.398 (8)	C151—C152	1.373 (8)
C352—C353	1.418 (7)	C152—C153	1.431 (7)
C352—C367	1.512 (8)	C152—C167	1.509 (8)
C353—H353	0.9500	C153—H153	0.9500
C353—C354	1.363 (9)	C153—C154	1.350 (9)
C354—H354	0.9500	C154—H154	0.9500
C354—C355	1.400 (9)	C154—C155	1.418 (9)
C355—C356	1.432 (9)	C155—C156	1.413 (8)
C356—H356	0.9500	C156—H156	0.9500
C356—C357	1.353 (10)	C156—C157	1.385 (9)
C357—H357	0.9500	C157—H157	0.9500
C357—C358	1.412 (8)	C157—C158	1.404 (7)
C358—C359	1.385 (8)	C158—C159	1.394 (8)
C358—C361	1.531 (10)	C158—C161	1.517 (8)
C359—H359	0.9500	C159—H159	0.9500
C361—H361	1.0000	C161—H161	1.0000
C361—C362	1.510 (11)	C161—C162	1.525 (8)
C361—C366	1.537 (10)	C161—C166	1.517 (10)
C362—H36A	0.9900	C162—H16C	0.9900
C362—H36B	0.9900	C162—H16D	0.9900
C362—C363	1.542 (12)	C162—C163	1.528 (9)
C363—H36C	0.9900	C163—H16E	0.9900
C363—H36D	0.9900	C163—H16F	0.9900
C363—C364	1.550 (12)	C163—C164	1.526 (11)
C364—H36E	0.9900	C164—H16G	0.9900
C364—H36F	0.9900	C164—H16H	0.9900
C364—C365	1.537 (12)	C164—C165	1.531 (13)
C365—H36G	0.9900	C165—H16I	0.9900
C365—H36H	0.9900	C165—H16J	0.9900
C365—C366	1.496 (11)	C165—C166	1.533 (11)
C366—H36I	0.9900	C166—H16K	0.9900
C366—H36J	0.9900	C166—H16L	0.9900
C367—H367	1.0000	C167—H167	1.0000
C367—C368	1.545 (8)	C167—C168	1.531 (8)
C367—C372	1.545 (9)	C167—C172	1.551 (9)
C368—H36K	0.9900	C168—H16M	0.9900
C368—H36L	0.9900	C168—H16N	0.9900
C368—C369	1.516 (8)	C168—C169	1.516 (10)
C369—H36M	0.9900	C169—H16O	0.9900
C369—H36N	0.9900	C169—H16P	0.9900
C369—C370	1.508 (9)	C169—C170	1.533 (11)
C370—H37A	0.9900	C170—H17C	0.9900
C370—H37B	0.9900	C170—H17D	0.9900
C370—C371	1.520 (10)	C170—C171	1.530 (10)
C371—H37C	0.9900	C171—H17E	0.9900
C371—H37D	0.9900	C171—H17F	0.9900
C371—C372	1.537 (9)	C171—C172	1.505 (8)
C372—H37E	0.9900	C172—H17G	0.9900
C372—H37F	0.9900	C172—H17H	0.9900
			
C211—Ir2—Cl2	90.41 (16)	C11—Ir1—Cl1	90.15 (16)
C211—Ir2—C301	151.6 (2)	C11—Ir1—C101	163.8 (2)
C211—Ir2—C302	172.0 (2)	C11—Ir1—C102	158.7 (2)
C211—Ir2—C303	97.0 (3)	C11—Ir1—C103	90.6 (2)
C211—Ir2—C304	93.4 (3)	C11—Ir1—C104	96.9 (2)
C301—Ir2—Cl2	87.81 (17)	C101—Ir1—Cl1	87.58 (16)
C301—Ir2—C302	36.3 (2)	C101—Ir1—C102	37.4 (2)
C302—Ir2—Cl2	88.36 (17)	C102—Ir1—Cl1	90.69 (16)
C303—Ir2—Cl2	155.7 (2)	C103—Ir1—Cl1	161.60 (18)
C303—Ir2—C301	96.2 (2)	C103—Ir1—C101	96.5 (3)
C303—Ir2—C302	81.1 (3)	C103—Ir1—C102	82.0 (2)
C303—Ir2—C304	38.2 (3)	C104—Ir1—Cl1	159.30 (18)
C304—Ir2—Cl2	164.58 (19)	C104—Ir1—C101	80.3 (2)
C304—Ir2—C301	81.7 (3)	C104—Ir1—C102	89.7 (2)
C304—Ir2—C302	89.8 (3)	C104—Ir1—C103	38.3 (3)
C211—N212—C213	109.3 (4)	C11—N12—C13	110.9 (4)
C211—N212—C321	126.0 (5)	C11—N12—C121	123.6 (5)
C321—N212—C213	119.0 (4)	C121—N12—C13	123.3 (4)
C211—N215—C214	108.8 (4)	C11—N15—C14	110.4 (4)
C211—N215—C351	126.6 (5)	C11—N15—C151	129.3 (5)
C351—N215—C214	122.8 (4)	C151—N15—C14	119.8 (4)
N212—C211—Ir2	129.3 (4)	N12—C11—Ir1	124.8 (4)
N215—C211—Ir2	122.9 (4)	N15—C11—Ir1	127.9 (4)
N215—C211—N212	107.8 (5)	N15—C11—N12	107.3 (5)
N212—C213—H213	107.6	N12—C13—H13	107.4
N212—C213—C214	100.0 (4)	N12—C13—C14	100.4 (4)
N212—C213—C216	120.1 (4)	N12—C13—C16	121.8 (4)
C214—C213—H213	107.6	C14—C13—H13	107.4
C214—C213—C216	113.0 (5)	C16—C13—H13	107.4
C216—C213—H213	107.6	C16—C13—C14	111.5 (5)
N215—C214—C213	101.2 (5)	N15—C14—C13	100.1 (4)
N215—C214—H214	107.3	N15—C14—H14	107.9
N215—C214—C219	122.8 (4)	N15—C14—C19	121.4 (4)
C213—C214—H214	107.3	C13—C14—H14	107.9
C213—C214—C219	110.0 (4)	C19—C14—C13	111.0 (4)
C219—C214—H214	107.3	C19—C14—H14	107.9
C213—C216—H21A	110.5	C13—C16—H16A	110.7
C213—C216—H21B	110.5	C13—C16—H16B	110.7
C213—C216—C217	106.0 (4)	C13—C16—C17	105.3 (4)
H21A—C216—H21B	108.7	H16A—C16—H16B	108.8
C217—C216—H21A	110.5	C17—C16—H16A	110.7
C217—C216—H21B	110.5	C17—C16—H16B	110.7
C216—C217—H21C	109.0	C16—C17—H17A	109.1
C216—C217—H21D	109.0	C16—C17—H17B	109.1
C216—C217—C218	112.7 (5)	H17A—C17—H17B	107.8
H21C—C217—H21D	107.8	C18—C17—C16	112.4 (5)
C218—C217—H21C	109.0	C18—C17—H17A	109.1
C218—C217—H21D	109.0	C18—C17—H17B	109.1
C217—C218—H21E	108.9	C17—C18—H18A	108.8
C217—C218—H21F	108.9	C17—C18—H18B	108.8
H21E—C218—H21F	107.7	C17—C18—C19	113.9 (4)
C219—C218—C217	113.5 (5)	H18A—C18—H18B	107.7
C219—C218—H21E	108.9	C19—C18—H18A	108.8
C219—C218—H21F	108.9	C19—C18—H18B	108.8
C214—C219—C218	106.9 (4)	C14—C19—C18	105.5 (4)
C214—C219—H21G	110.3	C14—C19—H19A	110.6
C214—C219—H21H	110.3	C14—C19—H19B	110.6
C218—C219—H21G	110.3	C18—C19—H19A	110.6
C218—C219—H21H	110.3	C18—C19—H19B	110.6
H21G—C219—H21H	108.6	H19A—C19—H19B	108.8
Ir2—C301—H301	112.8	Ir1—C101—H101	113.1
C302—C301—Ir2	74.4 (4)	C102—C101—Ir1	71.3 (3)
C302—C301—H301	112.8	C102—C101—H101	113.1
C302—C301—C306	128.0 (6)	C102—C101—C106	127.2 (5)
C306—C301—Ir2	108.9 (4)	C106—C101—Ir1	111.3 (4)
C306—C301—H301	112.8	C106—C101—H101	113.1
Ir2—C302—H302	115.0	Ir1—C102—H102	114.8
C301—C302—Ir2	69.3 (4)	C101—C102—Ir1	71.2 (3)
C301—C302—H302	115.0	C101—C102—H102	114.8
C301—C302—C307	122.5 (6)	C101—C102—C107	121.7 (5)
C307—C302—Ir2	111.4 (4)	C107—C102—Ir1	111.8 (4)
C307—C302—H302	115.0	C107—C102—H102	114.8
Ir2—C303—H303	113.5	Ir1—C103—H103	113.8
C304—C303—Ir2	71.0 (4)	C104—C103—Ir1	70.6 (4)
C304—C303—H303	113.5	C104—C103—H103	113.8
C304—C303—C308	124.9 (6)	C104—C103—C108	125.2 (6)
C308—C303—Ir2	113.1 (5)	C108—C103—Ir1	111.8 (4)
C308—C303—H303	113.5	C108—C103—H103	113.8
Ir2—C304—H304	114.0	Ir1—C104—H104	113.2
C303—C304—Ir2	70.8 (4)	C103—C104—Ir1	71.1 (4)
C303—C304—H304	114.0	C103—C104—H104	113.2
C303—C304—C305	124.9 (7)	C103—C104—C105	124.6 (6)
C305—C304—Ir2	111.4 (5)	C105—C104—Ir1	114.7 (4)
C305—C304—H304	114.0	C105—C104—H104	113.2
C304—C305—H30A	108.4	C104—C105—H10A	108.9
C304—C305—H30B	108.4	C104—C105—H10B	108.9
H30A—C305—H30B	107.5	H10A—C105—H10B	107.8
C306—C305—C304	115.5 (6)	C106—C105—C104	113.2 (5)
C306—C305—H30A	108.4	C106—C105—H10A	108.9
C306—C305—H30B	108.4	C106—C105—H10B	108.9
C301—C306—H30C	109.2	C101—C106—C105	112.1 (5)
C301—C306—H30D	109.2	C101—C106—H10C	109.2
C305—C306—C301	112.2 (6)	C101—C106—H10D	109.2
C305—C306—H30C	109.2	C105—C106—H10C	109.2
C305—C306—H30D	109.2	C105—C106—H10D	109.2
H30C—C306—H30D	107.9	H10C—C106—H10D	107.9
C302—C307—H30E	108.9	C102—C107—H10E	108.9
C302—C307—H30F	108.9	C102—C107—H10F	108.9
C302—C307—C308	113.2 (5)	C102—C107—C108	113.4 (5)
H30E—C307—H30F	107.7	H10E—C107—H10F	107.7
C308—C307—H30E	108.9	C108—C107—H10E	108.9
C308—C307—H30F	108.9	C108—C107—H10F	108.9
C303—C308—C307	113.2 (5)	C103—C108—C107	112.0 (5)
C303—C308—H30G	108.9	C103—C108—H10G	109.2
C303—C308—H30H	108.9	C103—C108—H10H	109.2
C307—C308—H30G	108.9	C107—C108—H10G	109.2
C307—C308—H30H	108.9	C107—C108—H10H	109.2
H30G—C308—H30H	107.8	H10G—C108—H10H	107.9
C325—C320—C321	118.6 (6)	C125—C120—C121	118.5 (5)
C329—C320—C321	123.3 (5)	C125—C120—C129	117.5 (5)
C329—C320—C325	118.1 (5)	C129—C120—C121	124.0 (5)
C320—C321—N212	116.8 (5)	C120—C121—N12	117.3 (5)
C322—C321—N212	121.5 (5)	C122—C121—N12	121.4 (5)
C322—C321—C320	121.3 (5)	C122—C121—C120	121.1 (5)
C321—C322—C323	118.4 (5)	C121—C122—C123	118.2 (5)
C321—C322—C337	123.8 (5)	C121—C122—C137	124.2 (5)
C323—C322—C337	117.6 (5)	C123—C122—C137	117.6 (5)
C322—C323—H323	119.1	C122—C123—H123	119.1
C324—C323—C322	121.8 (6)	C124—C123—C122	121.8 (6)
C324—C323—H323	119.1	C124—C123—H123	119.1
C323—C324—H324	119.7	C123—C124—H124	119.6
C323—C324—C325	120.6 (5)	C123—C124—C125	120.7 (5)
C325—C324—H324	119.7	C125—C124—H124	119.6
C324—C325—C320	119.3 (5)	C124—C125—C120	118.9 (5)
C324—C325—C326	122.0 (5)	C126—C125—C120	119.6 (6)
C326—C325—C320	118.7 (6)	C126—C125—C124	121.3 (5)
C325—C326—H326	119.6	C125—C126—H126	119.3
C327—C326—C325	120.9 (6)	C127—C126—C125	121.4 (5)
C327—C326—H326	119.6	C127—C126—H126	119.3
C326—C327—H327	119.1	C126—C127—H127	119.8
C326—C327—C328	121.8 (6)	C126—C127—C128	120.4 (6)
C328—C327—H327	119.1	C128—C127—H127	119.8
C327—C328—C331	118.6 (5)	C127—C128—C131	118.9 (5)
C329—C328—C327	118.5 (6)	C129—C128—C127	119.0 (6)
C329—C328—C331	122.8 (5)	C129—C128—C131	122.1 (5)
C320—C329—H329	119.0	C120—C129—H129	119.0
C328—C329—C320	121.9 (5)	C128—C129—C120	122.0 (5)
C328—C329—H329	119.0	C128—C129—H129	119.0
C328—C331—H331	107.6	C128—C131—H131	107.8
C328—C331—C332	112.3 (6)	C128—C131—C132	112.2 (5)
C328—C331—C336	112.5 (7)	C128—C131—C136	110.7 (5)
C332—C331—H331	107.6	C132—C131—H131	107.8
C332—C331—C336	109.1 (7)	C132—C131—C136	110.3 (5)
C336—C331—H331	107.6	C136—C131—H131	107.8
C331—C332—H33A	109.9	C131—C132—H13A	109.1
C331—C332—H33B	109.9	C131—C132—H13B	109.1
C331—C332—C333	108.9 (9)	C131—C132—C133	112.6 (6)
H33A—C332—H33B	108.3	H13A—C132—H13B	107.8
C333—C332—H33A	109.9	C133—C132—H13A	109.1
C333—C332—H33B	109.9	C133—C132—H13B	109.1
C332—C333—H33C	109.1	C132—C133—H13C	109.3
C332—C333—H33D	109.1	C132—C133—H13D	109.3
H33C—C333—H33D	107.9	H13C—C133—H13D	107.9
C334—C333—C332	112.3 (10)	C134—C133—C132	111.8 (6)
C334—C333—H33C	109.1	C134—C133—H13C	109.3
C334—C333—H33D	109.1	C134—C133—H13D	109.3
C333—C334—H33E	109.3	C133—C134—H13E	109.3
C333—C334—H33F	109.3	C133—C134—H13F	109.3
H33E—C334—H33F	107.9	C133—C134—C135	111.5 (6)
C335—C334—C333	111.8 (10)	H13E—C134—H13F	108.0
C335—C334—H33E	109.3	C135—C134—H13E	109.3
C335—C334—H33F	109.3	C135—C134—H13F	109.3
C334—C335—H33G	109.4	C134—C135—H13G	109.4
C334—C335—H33H	109.4	C134—C135—H13H	109.4
C334—C335—C336	111.1 (10)	C134—C135—C136	111.0 (6)
H33G—C335—H33H	108.0	H13G—C135—H13H	108.0
C336—C335—H33G	109.4	C136—C135—H13G	109.4
C336—C335—H33H	109.4	C136—C135—H13H	109.4
C331—C336—H33I	109.8	C131—C136—H13I	109.0
C331—C336—H33J	109.8	C131—C136—H13J	109.0
C335—C336—C331	109.2 (9)	C135—C136—C131	113.1 (6)
C335—C336—H33I	109.8	C135—C136—H13I	109.0
C335—C336—H33J	109.8	C135—C136—H13J	109.0
H33I—C336—H33J	108.3	H13I—C136—H13J	107.8
C322—C337—H337	108.1	C122—C137—H137	108.1
C322—C337—C338	109.9 (5)	C122—C137—C138	114.1 (5)
C322—C337—C342	112.6 (5)	C122—C137—C142	110.2 (5)
C338—C337—H337	108.1	C138—C137—H137	108.1
C342—C337—H337	108.1	C142—C137—H137	108.1
C342—C337—C338	109.9 (5)	C142—C137—C138	108.1 (5)
C337—C338—H33K	109.4	C137—C138—H13K	109.4
C337—C338—H33L	109.4	C137—C138—H13L	109.4
H33K—C338—H33L	108.0	H13K—C138—H13L	108.0
C339—C338—C337	111.1 (5)	C139—C138—C137	111.1 (5)
C339—C338—H33K	109.4	C139—C138—H13K	109.4
C339—C338—H33L	109.4	C139—C138—H13L	109.4
C338—C339—H33M	109.0	C138—C139—H13M	109.2
C338—C339—H33N	109.0	C138—C139—H13N	109.2
H33M—C339—H33N	107.8	C138—C139—C140	112.0 (6)
C340—C339—C338	113.1 (6)	H13M—C139—H13N	107.9
C340—C339—H33M	109.0	C140—C139—H13M	109.2
C340—C339—H33N	109.0	C140—C139—H13N	109.2
C339—C340—H34A	109.3	C139—C140—H14A	109.5
C339—C340—H34B	109.3	C139—C140—H14B	109.5
C339—C340—C341	111.7 (7)	H14A—C140—H14B	108.1
H34A—C340—H34B	107.9	C141—C140—C139	110.7 (5)
C341—C340—H34A	109.3	C141—C140—H14A	109.5
C341—C340—H34B	109.3	C141—C140—H14B	109.5
C340—C341—H34C	109.5	C140—C141—H14C	109.3
C340—C341—H34D	109.5	C140—C141—H14D	109.3
C340—C341—C342	110.8 (6)	C140—C141—C142	111.6 (5)
H34C—C341—H34D	108.1	H14C—C141—H14D	108.0
C342—C341—H34C	109.5	C142—C141—H14C	109.3
C342—C341—H34D	109.5	C142—C141—H14D	109.3
C337—C342—C341	110.8 (6)	C137—C142—H14E	109.4
C337—C342—H34E	109.5	C137—C142—H14F	109.4
C337—C342—H34F	109.5	C141—C142—C137	111.0 (5)
C341—C342—H34E	109.5	C141—C142—H14E	109.4
C341—C342—H34F	109.5	C141—C142—H14F	109.4
H34E—C342—H34F	108.1	H14E—C142—H14F	108.0
C355—C350—C351	118.2 (5)	C151—C150—C155	118.5 (5)
C359—C350—C351	123.4 (5)	C159—C150—C151	123.1 (5)
C359—C350—C355	118.4 (5)	C159—C150—C155	118.3 (5)
C350—C351—N215	118.9 (5)	C150—C151—N15	118.1 (5)
C352—C351—N215	120.3 (5)	C152—C151—N15	119.8 (5)
C352—C351—C350	120.8 (5)	C152—C151—C150	121.9 (5)
C351—C352—C353	117.8 (5)	C151—C152—C153	117.5 (5)
C351—C352—C367	124.4 (4)	C151—C152—C167	124.5 (5)
C353—C352—C367	117.6 (5)	C153—C152—C167	117.8 (5)
C352—C353—H353	118.8	C152—C153—H153	118.5
C354—C353—C352	122.5 (6)	C154—C153—C152	123.0 (6)
C354—C353—H353	118.8	C154—C153—H153	118.5
C353—C354—H354	119.8	C153—C154—H154	120.0
C353—C354—C355	120.4 (5)	C153—C154—C155	120.0 (5)
C355—C354—H354	119.8	C155—C154—H154	120.0
C350—C355—C356	118.3 (6)	C154—C155—C150	119.1 (5)
C354—C355—C350	119.8 (5)	C156—C155—C150	119.0 (6)
C354—C355—C356	121.9 (5)	C156—C155—C154	121.9 (5)
C355—C356—H356	119.6	C155—C156—H156	119.5
C357—C356—C355	120.9 (5)	C157—C156—C155	121.1 (5)
C357—C356—H356	119.6	C157—C156—H156	119.5
C356—C357—H357	119.0	C156—C157—H157	119.6
C356—C357—C358	122.0 (6)	C156—C157—C158	120.8 (5)
C358—C357—H357	119.0	C158—C157—H157	119.6
C357—C358—C361	119.9 (6)	C157—C158—C161	119.7 (5)
C359—C358—C357	118.0 (6)	C159—C158—C157	119.0 (5)
C359—C358—C361	122.0 (5)	C159—C158—C161	121.3 (5)
C350—C359—H359	118.8	C150—C159—H159	119.1
C358—C359—C350	122.3 (5)	C158—C159—C150	121.8 (5)
C358—C359—H359	118.8	C158—C159—H159	119.1
C358—C361—H361	108.3	C158—C161—H161	106.6
C358—C361—C366	111.4 (6)	C158—C161—C162	113.0 (6)
C362—C361—C358	110.8 (6)	C162—C161—H161	106.6
C362—C361—H361	108.3	C166—C161—C158	113.0 (6)
C362—C361—C366	109.8 (6)	C166—C161—H161	106.6
C366—C361—H361	108.3	C166—C161—C162	110.4 (5)
C361—C362—H36A	109.0	C161—C162—H16C	109.2
C361—C362—H36B	109.0	C161—C162—H16D	109.2
C361—C362—C363	112.9 (7)	C161—C162—C163	111.9 (5)
H36A—C362—H36B	107.8	H16C—C162—H16D	107.9
C363—C362—H36A	109.0	C163—C162—H16C	109.2
C363—C362—H36B	109.0	C163—C162—H16D	109.2
C362—C363—H36C	109.7	C162—C163—H16E	109.8
C362—C363—H36D	109.7	C162—C163—H16F	109.8
C362—C363—C364	109.8 (7)	H16E—C163—H16F	108.2
H36C—C363—H36D	108.2	C164—C163—C162	109.5 (6)
C364—C363—H36C	109.7	C164—C163—H16E	109.8
C364—C363—H36D	109.7	C164—C163—H16F	109.8
C363—C364—H36E	109.8	C163—C164—H16G	109.2
C363—C364—H36F	109.8	C163—C164—H16H	109.2
H36E—C364—H36F	108.2	C163—C164—C165	112.2 (6)
C365—C364—C363	109.6 (7)	H16G—C164—H16H	107.9
C365—C364—H36E	109.8	C165—C164—H16G	109.2
C365—C364—H36F	109.8	C165—C164—H16H	109.2
C364—C365—H36G	109.4	C164—C165—H16I	109.3
C364—C365—H36H	109.4	C164—C165—H16J	109.3
H36G—C365—H36H	108.0	C164—C165—C166	111.8 (6)
C366—C365—C364	111.1 (7)	H16I—C165—H16J	107.9
C366—C365—H36G	109.4	C166—C165—H16I	109.3
C366—C365—H36H	109.4	C166—C165—H16J	109.3
C361—C366—H36I	108.5	C161—C166—C165	111.2 (6)
C361—C366—H36J	108.5	C161—C166—H16K	109.4
C365—C366—C361	115.0 (7)	C161—C166—H16L	109.4
C365—C366—H36I	108.5	C165—C166—H16K	109.4
C365—C366—H36J	108.5	C165—C166—H16L	109.4
H36I—C366—H36J	107.5	H16K—C166—H16L	108.0
C352—C367—H367	108.7	C152—C167—H167	107.9
C352—C367—C368	110.2 (5)	C152—C167—C168	111.0 (5)
C352—C367—C372	113.8 (5)	C152—C167—C172	112.9 (5)
C368—C367—H367	108.7	C168—C167—H167	107.9
C368—C367—C372	106.7 (5)	C168—C167—C172	109.0 (5)
C372—C367—H367	108.7	C172—C167—H167	107.9
C367—C368—H36K	109.2	C167—C168—H16M	109.0
C367—C368—H36L	109.2	C167—C168—H16N	109.0
H36K—C368—H36L	107.9	H16M—C168—H16N	107.8
C369—C368—C367	112.0 (5)	C169—C168—C167	112.9 (5)
C369—C368—H36K	109.2	C169—C168—H16M	109.0
C369—C368—H36L	109.2	C169—C168—H16N	109.0
C368—C369—H36M	109.7	C168—C169—H16O	109.5
C368—C369—H36N	109.7	C168—C169—H16P	109.5
H36M—C369—H36N	108.2	C168—C169—C170	110.9 (6)
C370—C369—C368	110.0 (5)	H16O—C169—H16P	108.0
C370—C369—H36M	109.7	C170—C169—H16O	109.5
C370—C369—H36N	109.7	C170—C169—H16P	109.5
C369—C370—H37A	109.1	C169—C170—H17C	109.3
C369—C370—H37B	109.1	C169—C170—H17D	109.3
C369—C370—C371	112.4 (5)	H17C—C170—H17D	107.9
H37A—C370—H37B	107.9	C171—C170—C169	111.7 (6)
C371—C370—H37A	109.1	C171—C170—H17C	109.3
C371—C370—H37B	109.1	C171—C170—H17D	109.3
C370—C371—H37C	109.3	C170—C171—H17E	109.2
C370—C371—H37D	109.3	C170—C171—H17F	109.2
C370—C371—C372	111.4 (6)	H17E—C171—H17F	107.9
H37C—C371—H37D	108.0	C172—C171—C170	112.1 (6)
C372—C371—H37C	109.3	C172—C171—H17E	109.2
C372—C371—H37D	109.3	C172—C171—H17F	109.2
C367—C372—H37E	109.3	C167—C172—H17G	109.3
C367—C372—H37F	109.3	C167—C172—H17H	109.3
C371—C372—C367	111.5 (5)	C171—C172—C167	111.7 (6)
C371—C372—H37E	109.3	C171—C172—H17G	109.3
C371—C372—H37F	109.3	C171—C172—H17H	109.3
H37E—C372—H37F	108.0	H17G—C172—H17H	107.9
			
Ir2—C301—C302—C307	102.8 (6)	Ir1—C101—C102—C107	104.6 (5)
Ir2—C301—C306—C305	−35.4 (6)	Ir1—C101—C106—C105	−32.2 (6)
Ir2—C302—C307—C308	−11.1 (7)	Ir1—C102—C107—C108	−10.7 (6)
Ir2—C303—C304—C305	103.2 (7)	Ir1—C103—C104—C105	107.5 (6)
Ir2—C303—C308—C307	−31.8 (7)	Ir1—C103—C108—C107	−33.2 (7)
Ir2—C304—C305—C306	−7.7 (8)	Ir1—C104—C105—C106	−9.5 (7)
N212—C213—C214—N215	−34.1 (5)	N12—C13—C14—N15	−30.9 (5)
N212—C213—C214—C219	−165.4 (5)	N12—C13—C14—C19	−160.3 (5)
N212—C213—C216—C217	−177.9 (5)	N12—C13—C16—C17	−179.8 (5)
N212—C321—C322—C323	173.5 (5)	N12—C121—C122—C123	−178.6 (5)
N212—C321—C322—C337	−1.0 (9)	N12—C121—C122—C137	4.2 (9)
N215—C214—C219—C218	−177.2 (5)	N15—C14—C19—C18	−176.6 (5)
N215—C351—C352—C353	173.6 (5)	N15—C151—C152—C153	−173.2 (5)
N215—C351—C352—C367	−11.0 (9)	N15—C151—C152—C167	3.2 (9)
C211—N212—C213—C214	29.7 (6)	C11—N12—C13—C14	26.5 (6)
C211—N212—C213—C216	153.9 (5)	C11—N12—C13—C16	150.0 (5)
C211—N212—C321—C320	−111.6 (6)	C11—N12—C121—C120	−91.0 (7)
C211—N212—C321—C322	74.6 (8)	C11—N12—C121—C122	93.9 (7)
C211—N215—C214—C213	30.1 (6)	C11—N15—C14—C13	28.8 (6)
C211—N215—C214—C219	153.0 (5)	C11—N15—C14—C19	151.1 (5)
C211—N215—C351—C350	79.9 (8)	C11—N15—C151—C150	103.9 (7)
C211—N215—C351—C352	−100.9 (7)	C11—N15—C151—C152	−82.2 (8)
C213—N212—C211—Ir2	170.6 (4)	C13—N12—C11—Ir1	171.3 (4)
C213—N212—C211—N215	−11.8 (6)	C13—N12—C11—N15	−9.2 (6)
C213—N212—C321—C320	97.9 (6)	C13—N12—C121—C120	107.3 (6)
C213—N212—C321—C322	−75.9 (7)	C13—N12—C121—C122	−67.9 (7)
C213—C214—C219—C218	−58.5 (6)	C13—C14—C19—C18	−59.7 (6)
C213—C216—C217—C218	53.1 (7)	C13—C16—C17—C18	54.0 (6)
C214—N215—C211—Ir2	165.8 (4)	C14—N15—C11—Ir1	166.2 (4)
C214—N215—C211—N212	−12.0 (6)	C14—N15—C11—N12	−13.2 (6)
C214—N215—C351—C350	−83.1 (7)	C14—N15—C151—C150	−66.7 (7)
C214—N215—C351—C352	96.1 (7)	C14—N15—C151—C152	107.1 (6)
C214—C213—C216—C217	−60.2 (6)	C14—C13—C16—C17	−61.6 (5)
C216—C213—C214—N215	−163.1 (5)	C16—C13—C14—N15	−161.3 (4)
C216—C213—C214—C219	65.6 (6)	C16—C13—C14—C19	69.3 (6)
C216—C217—C218—C219	−54.1 (7)	C16—C17—C18—C19	−53.1 (7)
C217—C218—C219—C214	53.9 (7)	C17—C18—C19—C14	53.4 (7)
C301—C302—C307—C308	−89.6 (8)	C101—C102—C107—C108	−91.5 (7)
C302—C301—C306—C305	49.2 (9)	C102—C101—C106—C105	50.0 (8)
C302—C307—C308—C303	27.8 (8)	C102—C107—C108—C103	28.7 (7)
C303—C304—C305—C306	−88.6 (9)	C103—C104—C105—C106	−92.8 (8)
C304—C303—C308—C307	50.6 (10)	C104—C103—C108—C107	47.9 (9)
C304—C305—C306—C301	28.9 (8)	C104—C105—C106—C101	27.4 (7)
C306—C301—C302—Ir2	−102.0 (7)	C106—C101—C102—Ir1	−103.0 (6)
C306—C301—C302—C307	0.8 (10)	C106—C101—C102—C107	1.5 (9)
C308—C303—C304—Ir2	−105.4 (7)	C108—C103—C104—Ir1	−103.5 (7)
C308—C303—C304—C305	−2.2 (12)	C108—C103—C104—C105	4.0 (11)
C320—C321—C322—C323	0.0 (9)	C120—C121—C122—C123	6.4 (9)
C320—C321—C322—C337	−174.5 (5)	C120—C121—C122—C137	−170.8 (5)
C320—C325—C326—C327	−0.1 (10)	C120—C125—C126—C127	−0.9 (10)
C321—N212—C211—Ir2	17.7 (8)	C121—N12—C11—Ir1	7.6 (8)
C321—N212—C211—N215	−164.6 (5)	C121—N12—C11—N15	−172.9 (5)
C321—N212—C213—C214	−175.3 (5)	C121—N12—C13—C14	−169.7 (5)
C321—N212—C213—C216	−51.1 (7)	C121—N12—C13—C16	−46.2 (7)
C321—C320—C325—C324	1.2 (9)	C121—C120—C125—C124	5.6 (9)
C321—C320—C325—C326	−177.5 (6)	C121—C120—C125—C126	−178.6 (6)
C321—C320—C329—C328	176.9 (6)	C121—C120—C129—C128	−179.7 (6)
C321—C322—C323—C324	0.7 (9)	C121—C122—C123—C124	1.7 (9)
C321—C322—C337—C338	104.5 (6)	C121—C122—C137—C138	−144.1 (6)
C321—C322—C337—C342	−132.6 (6)	C121—C122—C137—C142	94.1 (6)
C322—C323—C324—C325	−0.5 (10)	C122—C123—C124—C125	−6.0 (10)
C322—C337—C338—C339	−179.0 (5)	C122—C137—C138—C139	178.1 (5)
C322—C337—C342—C341	179.7 (4)	C122—C137—C142—C141	−176.0 (5)
C323—C322—C337—C338	−70.0 (7)	C123—C122—C137—C138	38.7 (7)
C323—C322—C337—C342	52.9 (7)	C123—C122—C137—C142	−83.1 (6)
C323—C324—C325—C320	−0.5 (9)	C123—C124—C125—C120	2.1 (9)
C323—C324—C325—C326	178.2 (6)	C123—C124—C125—C126	−173.6 (6)
C324—C325—C326—C327	−178.8 (6)	C124—C125—C126—C127	174.8 (6)
C325—C320—C321—N212	−174.7 (5)	C125—C120—C121—N12	174.8 (5)
C325—C320—C321—C322	−1.0 (9)	C125—C120—C121—C122	−10.0 (9)
C325—C320—C329—C328	−1.5 (9)	C125—C120—C129—C128	−1.2 (9)
C325—C326—C327—C328	−0.3 (10)	C125—C126—C127—C128	−2.8 (10)
C326—C327—C328—C329	−0.2 (10)	C126—C127—C128—C129	4.5 (10)
C326—C327—C328—C331	178.5 (6)	C126—C127—C128—C131	−175.9 (6)
C327—C328—C329—C320	1.2 (10)	C127—C128—C129—C120	−2.4 (9)
C327—C328—C331—C332	110.2 (7)	C127—C128—C131—C132	−51.6 (8)
C327—C328—C331—C336	−126.3 (7)	C127—C128—C131—C136	72.1 (7)
C328—C331—C332—C333	−179.2 (7)	C128—C131—C132—C133	172.2 (5)
C328—C331—C336—C335	173.8 (7)	C128—C131—C136—C135	−174.9 (5)
C329—C320—C321—N212	6.9 (9)	C129—C120—C121—N12	−6.7 (9)
C329—C320—C321—C322	−179.4 (6)	C129—C120—C121—C122	168.5 (6)
C329—C320—C325—C324	179.7 (6)	C129—C120—C125—C124	−172.9 (6)
C329—C320—C325—C326	1.0 (9)	C129—C120—C125—C126	2.8 (9)
C329—C328—C331—C332	−71.1 (9)	C129—C128—C131—C132	128.0 (6)
C329—C328—C331—C336	52.4 (10)	C129—C128—C131—C136	−108.2 (6)
C331—C328—C329—C320	−177.6 (6)	C131—C128—C129—C120	178.0 (6)
C331—C332—C333—C334	−52.8 (11)	C131—C132—C133—C134	−53.4 (7)
C332—C331—C336—C335	−61.0 (10)	C132—C131—C136—C135	−50.0 (7)
C332—C333—C334—C335	54.2 (13)	C132—C133—C134—C135	57.7 (7)
C333—C334—C335—C336	−59.2 (13)	C133—C134—C135—C136	−58.8 (8)
C334—C335—C336—C331	62.2 (12)	C134—C135—C136—C131	55.0 (7)
C336—C331—C332—C333	55.3 (9)	C136—C131—C132—C133	48.3 (7)
C337—C322—C323—C324	175.6 (6)	C137—C122—C123—C124	179.0 (6)
C337—C338—C339—C340	−55.3 (7)	C137—C138—C139—C140	57.6 (7)
C338—C337—C342—C341	−57.4 (6)	C138—C137—C142—C141	58.7 (6)
C338—C339—C340—C341	53.7 (7)	C138—C139—C140—C141	−53.9 (7)
C339—C340—C341—C342	−53.5 (8)	C139—C140—C141—C142	53.4 (7)
C340—C341—C342—C337	55.9 (7)	C140—C141—C142—C137	−57.3 (7)
C342—C337—C338—C339	56.5 (7)	C142—C137—C138—C139	−59.0 (6)
C350—C351—C352—C353	−7.3 (9)	C150—C151—C152—C153	0.5 (9)
C350—C351—C352—C367	168.2 (5)	C150—C151—C152—C167	176.8 (5)
C350—C355—C356—C357	3.9 (10)	C150—C155—C156—C157	−0.1 (9)
C351—N215—C211—Ir2	0.9 (8)	C151—N15—C11—Ir1	−5.1 (9)
C351—N215—C211—N212	−177.0 (5)	C151—N15—C11—N12	175.4 (5)
C351—N215—C214—C213	−164.2 (5)	C151—N15—C14—C13	−158.9 (5)
C351—N215—C214—C219	−41.4 (8)	C151—N15—C14—C19	−36.6 (7)
C351—C350—C355—C354	−3.1 (9)	C151—C150—C155—C154	0.0 (9)
C351—C350—C355—C356	179.2 (6)	C151—C150—C155—C156	177.7 (5)
C351—C350—C359—C358	178.9 (6)	C151—C150—C159—C158	−176.3 (5)
C351—C352—C353—C354	1.3 (9)	C151—C152—C153—C154	−0.1 (9)
C351—C352—C367—C368	−104.7 (6)	C151—C152—C167—C168	−109.7 (6)
C351—C352—C367—C372	135.4 (6)	C151—C152—C167—C172	127.5 (6)
C352—C353—C354—C355	3.8 (10)	C152—C153—C154—C155	−0.4 (10)
C352—C367—C368—C369	175.5 (5)	C152—C167—C168—C169	178.8 (5)
C352—C367—C372—C371	179.4 (5)	C152—C167—C172—C171	179.5 (4)
C353—C352—C367—C368	70.7 (7)	C153—C152—C167—C168	66.6 (7)
C353—C352—C367—C372	−49.1 (7)	C153—C152—C167—C172	−56.2 (7)
C353—C354—C355—C350	−2.8 (10)	C153—C154—C155—C150	0.4 (9)
C353—C354—C355—C356	174.9 (6)	C153—C154—C155—C156	−177.2 (6)
C354—C355—C356—C357	−173.8 (7)	C154—C155—C156—C157	177.6 (6)
C355—C350—C351—N215	−172.6 (5)	C155—C150—C151—N15	173.3 (5)
C355—C350—C351—C352	8.2 (9)	C155—C150—C151—C152	−0.4 (9)
C355—C350—C359—C358	0.9 (9)	C155—C150—C159—C158	2.0 (9)
C355—C356—C357—C358	−3.2 (11)	C155—C156—C157—C158	−0.5 (10)
C356—C357—C358—C359	1.3 (11)	C156—C157—C158—C159	1.7 (9)
C356—C357—C358—C361	177.8 (7)	C156—C157—C158—C161	−178.4 (6)
C357—C358—C359—C350	−0.1 (10)	C157—C158—C159—C150	−2.5 (9)
C357—C358—C361—C362	−67.3 (8)	C157—C158—C161—C162	127.4 (6)
C357—C358—C361—C366	55.1 (9)	C157—C158—C161—C166	−106.3 (6)
C358—C361—C362—C363	176.3 (6)	C158—C161—C162—C163	−173.8 (5)
C358—C361—C366—C365	−174.7 (6)	C158—C161—C166—C165	176.6 (5)
C359—C350—C351—N215	9.3 (9)	C159—C150—C151—N15	−8.5 (8)
C359—C350—C351—C352	−169.9 (6)	C159—C150—C151—C152	177.8 (6)
C359—C350—C355—C354	175.1 (6)	C159—C150—C155—C154	−178.3 (5)
C359—C350—C355—C356	−2.7 (9)	C159—C150—C155—C156	−0.6 (9)
C359—C358—C361—C362	109.0 (7)	C159—C158—C161—C162	−52.7 (8)
C359—C358—C361—C366	−128.5 (7)	C159—C158—C161—C166	73.6 (7)
C361—C358—C359—C350	−176.5 (6)	C161—C158—C159—C150	177.6 (6)
C361—C362—C363—C364	−57.8 (10)	C161—C162—C163—C164	−57.3 (8)
C362—C361—C366—C365	−51.6 (8)	C162—C161—C166—C165	−55.7 (7)
C362—C363—C364—C365	57.7 (10)	C162—C163—C164—C165	54.6 (8)
C363—C364—C365—C366	−56.1 (9)	C163—C164—C165—C166	−53.5 (8)
C364—C365—C366—C361	54.1 (8)	C164—C165—C166—C161	53.6 (8)
C366—C361—C362—C363	52.9 (9)	C166—C161—C162—C163	58.5 (7)
C367—C352—C353—C354	−174.5 (6)	C167—C152—C153—C154	−176.6 (6)
C367—C368—C369—C370	59.6 (6)	C167—C168—C169—C170	55.6 (8)
C368—C367—C372—C371	57.6 (6)	C168—C167—C172—C171	55.7 (6)
C368—C369—C370—C371	−54.7 (7)	C168—C169—C170—C171	−53.0 (8)
C369—C370—C371—C372	53.1 (7)	C169—C170—C171—C172	53.8 (8)
C370—C371—C372—C367	−55.4 (7)	C170—C171—C172—C167	−55.5 (7)
C372—C367—C368—C369	−60.4 (6)	C172—C167—C168—C169	−56.2 (7)

### [µ2-(Ra,Sa,3aR,7aR)-1,3-bis(2,7-Dicyclohexylnaphthalen-1-yl)octahydro-1H-benzo[d]imidazolidin-2-ylidene]chlorido(η4-1,5-cyclooctadiene)iridium dichloromethane monosolvate (I) Hydrogen-bond geometry (Å, º)

**Table:**

*D*—H···*A*	*D*—H	H···*A*	*D*···*A*	*D*—H···*A*
C17—H17*A*···Cl1^i^	0.99	2.79	3.773 (6)	171
C353—H353···Cl1	0.95	2.80	3.650 (6)	149
C372—H37*E*···Cl2	0.99	2.56	3.422 (6)	146

Symmetry code: (i) *x*, *y*−1, *z*.

### [µ2-(Sa,Sa,3aR,7aR)-1,3-bis(2,7-Dicyclohexylnaphthalen-1-yl)octahydro-1H-benzo[d]imidazolidin-2-ylidene]chlorido(η4-1,5-cyclooctadiene)iridium (II) Crystal data

**Table:**

[Ir(C_51_H_64_N_2_)Cl(C_8_H_12_)]	*F*(000) = 2152
*M**_r_* = 1040.86	*D*_x_ = 1.405 Mg m^−^^3^
Orthorhombic, *P*2_1_2_1_2_1_	Cu *K*α radiation, λ = 1.54184 Å
Hall symbol: P 2ac 2ab	Cell parameters from 10786 reflections
*a* = 16.1663 (4) Å	θ = 3.6–67.3°
*b* = 17.2811 (4) Å	µ = 6.04 mm^−^^1^
*c* = 17.6110 (3) Å	*T* = 100 K
*V* = 4920.01 (19) Å^3^	Needle, yellow
*Z* = 4	0.25 × 0.03 × 0.02 mm

### [µ2-(Sa,Sa,3aR,7aR)-1,3-bis(2,7-Dicyclohexylnaphthalen-1-yl)octahydro-1H-benzo[d]imidazolidin-2-ylidene]chlorido(η4-1,5-cyclooctadiene)iridium (II) Data collection

**Table:**

Oxford Diffraction Gemini diffractometer	8782 independent reflections
Radiation source: sealed X-ray tube, Enhance Ultra (Cu) X-ray Source	8164 reflections with *I* > 2σ(*I*)
Mirror monochromator	*R*_int_ = 0.056
Detector resolution: 10.4738 pixels mm^-1^	θ_max_ = 67.7°, θ_min_ = 3.6°
ω scans	*h* = −19→17
Absorption correction: analytical CrysAlisPro (Rigaku OD, 2019)	*k* = −19→20
*T*_min_ = 0.50, *T*_max_ = 0.90	*l* = −19→20
26296 measured reflections	

### [µ2-(Sa,Sa,3aR,7aR)-1,3-bis(2,7-Dicyclohexylnaphthalen-1-yl)octahydro-1H-benzo[d]imidazolidin-2-ylidene]chlorido(η4-1,5-cyclooctadiene)iridium (II) Refinement

**Table:**

Refinement on *F*^2^	Hydrogen site location: inferred from neighbouring sites
Least-squares matrix: full	H-atom parameters constrained
*R*[*F*^2^ > 2σ(*F*^2^)] = 0.039	*w* = 1/[σ^2^(*F*_o_^2^) + (0.0588*P*)^2^] where *P* = (*F*_o_^2^ + 2*F*_c_^2^)/3
*wR*(*F*^2^) = 0.098	(Δ/σ)_max_ = 0.001
*S* = 1.04	Δρ_max_ = 2.41 e Å^−^^3^
8782 reflections	Δρ_min_ = −0.91 e Å^−^^3^
568 parameters	Absolute structure: Flack *x* determined using 3426 quotients [(*I*^+^)-(*I*^-^)]/[(*I*^+^)+(*I*^-^)] (Parsons *et al.*, 2013)
0 restraints	Absolute structure parameter: −0.046 (7)

### [µ2-(Sa,Sa,3aR,7aR)-1,3-bis(2,7-Dicyclohexylnaphthalen-1-yl)octahydro-1H-benzo[d]imidazolidin-2-ylidene]chlorido(η4-1,5-cyclooctadiene)iridium (II) Special details

**Table:**

Geometry. All esds (except the esd in the dihedral angle between two l.s. planes) are estimated using the full covariance matrix. The cell esds are taken into account individually in the estimation of esds in distances, angles and torsion angles; correlations between esds in cell parameters are only used when they are defined by crystal symmetry. An approximate (isotropic) treatment of cell esds is used for estimating esds involving l.s. planes.
Refinement. One reflection with poor agreement was omitted from the refinement.

### [µ2-(Sa,Sa,3aR,7aR)-1,3-bis(2,7-Dicyclohexylnaphthalen-1-yl)octahydro-1H-benzo[d]imidazolidin-2-ylidene]chlorido(η4-1,5-cyclooctadiene)iridium (II) Fractional atomic coordinates and isotropic or equivalent isotropic displacement parameters (Å^2^)

**Table:**

	*x*	*y*	*z*	*U*_iso_*/*U*_eq_	
Ir1	0.54235 (2)	0.59059 (2)	0.39700 (2)	0.02181 (11)	
Cl1	0.41450 (11)	0.58045 (12)	0.46083 (10)	0.0271 (4)	
C101	0.4761 (6)	0.6278 (5)	0.2922 (4)	0.0294 (19)	
H101	0.430379	0.596679	0.30705	0.035*	
C102	0.4939 (5)	0.6920 (5)	0.3383 (5)	0.0265 (18)	
H102	0.457621	0.701343	0.379729	0.032*	
C103	0.6542 (5)	0.6303 (5)	0.3450 (5)	0.0260 (17)	
H103	0.676855	0.631076	0.394737	0.031*	
C104	0.6311 (5)	0.5567 (5)	0.3138 (5)	0.0267 (17)	
H104	0.642458	0.511952	0.343342	0.032*	
C105	0.5902 (5)	0.5451 (5)	0.2382 (5)	0.0279 (18)	
H10A	0.566757	0.492262	0.236247	0.033*	
H10B	0.632778	0.548944	0.197889	0.033*	
C106	0.5221 (5)	0.6028 (5)	0.2214 (5)	0.0302 (19)	
H10C	0.482453	0.57934	0.185318	0.036*	
H10D	0.546481	0.648976	0.196777	0.036*	
C107	0.5648 (6)	0.7477 (5)	0.3284 (5)	0.034 (2)	
H10E	0.574512	0.775328	0.376803	0.041*	
H10F	0.549921	0.78662	0.289465	0.041*	
C108	0.6449 (6)	0.7066 (5)	0.3043 (5)	0.0305 (19)	
H10G	0.643981	0.697551	0.248752	0.037*	
H10H	0.692941	0.740043	0.315985	0.037*	
C11	0.5961 (5)	0.5311 (5)	0.4844 (4)	0.0213 (16)	
N12	0.6073 (4)	0.5585 (4)	0.5563 (4)	0.0214 (14)	
C121	0.5956 (5)	0.6363 (5)	0.5851 (4)	0.0215 (16)	
C122	0.6570 (5)	0.6908 (5)	0.5780 (4)	0.0246 (17)	
C123	0.6459 (5)	0.7638 (5)	0.6144 (5)	0.0258 (17)	
H123	0.68478	0.803936	0.605349	0.031*	
C124	0.5811 (5)	0.7774 (5)	0.6615 (4)	0.0256 (16)	
H124	0.576518	0.825906	0.686505	0.031*	
C125	0.5207 (5)	0.7200 (5)	0.6737 (4)	0.0247 (17)	
C126	0.4548 (6)	0.7297 (4)	0.7271 (4)	0.0269 (16)	
H126	0.450872	0.776472	0.755194	0.032*	
C127	0.3973 (5)	0.6726 (5)	0.7384 (4)	0.0248 (17)	
H127	0.355672	0.679523	0.775885	0.03*	
C128	0.3989 (5)	0.6034 (5)	0.6952 (5)	0.0266 (17)	
C129	0.4613 (5)	0.5939 (5)	0.6433 (4)	0.0227 (14)	
H129	0.462154	0.548212	0.613328	0.027*	
C120	0.5251 (5)	0.6499 (4)	0.6326 (4)	0.0204 (16)	
C161	0.7386 (5)	0.6754 (5)	0.5391 (4)	0.0233 (16)	
H161	0.733987	0.624889	0.511759	0.028*	
C162	0.8085 (5)	0.6678 (5)	0.5983 (5)	0.0273 (16)	
H16A	0.816275	0.717833	0.624684	0.033*	
H16B	0.793018	0.628495	0.636703	0.033*	
C163	0.8895 (5)	0.6439 (6)	0.5597 (5)	0.0329 (19)	
H16C	0.934339	0.64292	0.597934	0.04*	
H16D	0.883456	0.590944	0.538973	0.04*	
C164	0.9135 (6)	0.6987 (5)	0.4957 (5)	0.0330 (19)	
H16E	0.961411	0.676866	0.467991	0.04*	
H16F	0.930811	0.748878	0.517712	0.04*	
C165	0.8430 (6)	0.7126 (6)	0.4397 (5)	0.034 (2)	
H16G	0.832436	0.664679	0.410497	0.041*	
H16H	0.859068	0.753699	0.403469	0.041*	
C166	0.7638 (5)	0.7368 (5)	0.4814 (4)	0.0284 (18)	
H16I	0.773054	0.786637	0.507716	0.034*	
H16J	0.718559	0.744093	0.444179	0.034*	
C171	0.3316 (5)	0.5443 (5)	0.7060 (5)	0.0287 (18)	
H171	0.316697	0.54436	0.761102	0.034*	
C172	0.3565 (5)	0.4618 (5)	0.6853 (5)	0.0299 (18)	
H17C	0.40551	0.446625	0.715605	0.036*	
H17D	0.372039	0.459751	0.631004	0.036*	
C173	0.2864 (5)	0.4044 (6)	0.7001 (5)	0.0334 (18)	
H17E	0.303911	0.351856	0.684549	0.04*	
H17F	0.27361	0.403168	0.755108	0.04*	
C174	0.2094 (6)	0.4279 (6)	0.6559 (5)	0.038 (2)	
H17G	0.220897	0.423876	0.600786	0.045*	
H17H	0.163791	0.391721	0.668042	0.045*	
C175	0.1828 (6)	0.5096 (6)	0.6744 (6)	0.038 (2)	
H17I	0.164534	0.512181	0.728051	0.045*	
H17J	0.135308	0.524168	0.641923	0.045*	
C176	0.2538 (6)	0.5670 (5)	0.6618 (6)	0.035 (2)	
H17K	0.267029	0.569244	0.606981	0.042*	
H17L	0.235865	0.619238	0.677952	0.042*	
C13	0.6188 (5)	0.4929 (4)	0.6090 (5)	0.0236 (15)	
H13	0.562656	0.47308	0.622637	0.028*	
C14	0.6579 (5)	0.4347 (5)	0.5573 (4)	0.0214 (16)	
H14	0.718056	0.447535	0.55329	0.026*	
N15	0.6182 (4)	0.4564 (4)	0.4847 (4)	0.0223 (14)	
C151	0.6255 (5)	0.4036 (5)	0.4216 (4)	0.0238 (15)	
C152	0.5576 (4)	0.3625 (4)	0.3968 (5)	0.0236 (15)	
C153	0.5708 (5)	0.3055 (5)	0.3389 (5)	0.0282 (18)	
H153	0.524944	0.276669	0.320479	0.034*	
C154	0.6476 (6)	0.2917 (5)	0.3097 (5)	0.0296 (18)	
H154	0.654164	0.253125	0.271751	0.035*	
C155	0.7167 (5)	0.3329 (5)	0.3344 (4)	0.0260 (17)	
C156	0.7967 (5)	0.3190 (5)	0.3058 (5)	0.0275 (17)	
H156	0.804137	0.281127	0.267303	0.033*	
C157	0.8641 (6)	0.3590 (5)	0.3324 (5)	0.0287 (18)	
H157	0.917681	0.346593	0.313805	0.034*	
C158	0.8550 (5)	0.4179 (5)	0.3865 (4)	0.0246 (16)	
C159	0.7772 (5)	0.4340 (5)	0.4133 (4)	0.0254 (18)	
H159	0.770588	0.475338	0.448369	0.031*	
C150	0.7063 (5)	0.3917 (4)	0.3908 (5)	0.0235 (15)	
C181	0.4720 (5)	0.3702 (5)	0.4297 (4)	0.0262 (17)	
H181	0.47334	0.412284	0.468645	0.031*	
C182	0.4456 (5)	0.2949 (5)	0.4694 (5)	0.0307 (18)	
H18A	0.447569	0.2517	0.432519	0.037*	
H18B	0.485077	0.283093	0.510813	0.037*	
C183	0.3586 (6)	0.3007 (6)	0.5023 (5)	0.036 (2)	
H18C	0.357774	0.340291	0.542917	0.043*	
H18D	0.342937	0.250436	0.525169	0.043*	
C184	0.2955 (6)	0.3224 (6)	0.4408 (6)	0.036 (2)	
H18E	0.293029	0.280959	0.402041	0.044*	
H18F	0.239923	0.327664	0.463787	0.044*	
C185	0.3202 (5)	0.3985 (6)	0.4032 (5)	0.0344 (19)	
H18G	0.318776	0.440648	0.441306	0.041*	
H18H	0.280079	0.411315	0.362623	0.041*	
C186	0.4080 (5)	0.3927 (5)	0.3689 (5)	0.0299 (19)	
H18I	0.4082	0.353415	0.327973	0.036*	
H18J	0.423522	0.443075	0.34628	0.036*	
C191	0.9290 (6)	0.4584 (5)	0.4195 (5)	0.0297 (19)	
H191	0.908722	0.504741	0.448049	0.036*	
C192	0.9725 (6)	0.4040 (9)	0.4770 (6)	0.052 (3)	
H19A	0.932509	0.38905	0.516898	0.063*	
H19B	0.989976	0.356292	0.450308	0.063*	
C193	1.0474 (7)	0.4407 (8)	0.5141 (6)	0.058 (3)	
H19C	1.074621	0.402467	0.547638	0.069*	
H19D	1.029534	0.485004	0.545713	0.069*	
C194	1.1091 (6)	0.4688 (7)	0.4545 (6)	0.043 (2)	
H19E	1.155584	0.49554	0.48007	0.052*	
H19F	1.131915	0.423725	0.426814	0.052*	
C195	1.0687 (5)	0.5234 (5)	0.3988 (6)	0.0344 (18)	
H19G	1.109179	0.53805	0.359187	0.041*	
H19H	1.051474	0.571068	0.425642	0.041*	
C196	0.9931 (6)	0.4862 (6)	0.3613 (5)	0.038 (2)	
H19I	1.011386	0.441661	0.330211	0.045*	
H19J	0.966896	0.524315	0.326902	0.045*	
C16	0.6671 (5)	0.5007 (5)	0.6816 (5)	0.0276 (17)	
H16K	0.640431	0.538744	0.715698	0.033*	
H16L	0.724238	0.518043	0.670861	0.033*	
C17	0.6677 (6)	0.4197 (5)	0.7185 (4)	0.0304 (18)	
H17A	0.703986	0.420942	0.763782	0.037*	
H17B	0.611018	0.40712	0.735927	0.037*	
C18	0.6974 (5)	0.3558 (5)	0.6653 (5)	0.0273 (17)	
H18K	0.690463	0.305221	0.690872	0.033*	
H18L	0.757209	0.363156	0.655391	0.033*	
C19	0.6508 (5)	0.3541 (5)	0.5886 (4)	0.0261 (17)	
H19K	0.676383	0.316189	0.553631	0.031*	
H19L	0.592066	0.339894	0.596329	0.031*	

### [µ2-(Sa,Sa,3aR,7aR)-1,3-bis(2,7-Dicyclohexylnaphthalen-1-yl)octahydro-1H-benzo[d]imidazolidin-2-ylidene]chlorido(η4-1,5-cyclooctadiene)iridium (II) Atomic displacement parameters (Å^2^)

**Table:**

	*U*^11^	*U*^22^	*U*^33^	*U*^12^	*U*^13^	*U*^23^
Ir1	0.02523 (16)	0.02132 (16)	0.01887 (16)	0.00163 (14)	−0.00061 (13)	0.00003 (13)
Cl1	0.0241 (9)	0.0308 (11)	0.0264 (9)	0.0000 (8)	0.0015 (7)	0.0006 (8)
C101	0.037 (5)	0.033 (4)	0.018 (4)	0.007 (4)	−0.006 (3)	0.008 (3)
C102	0.036 (4)	0.016 (4)	0.028 (4)	0.012 (3)	−0.001 (3)	0.012 (3)
C103	0.024 (4)	0.034 (5)	0.021 (4)	−0.006 (3)	0.005 (3)	0.003 (3)
C104	0.029 (4)	0.026 (4)	0.026 (4)	0.006 (3)	0.009 (4)	0.002 (3)
C105	0.032 (5)	0.028 (5)	0.023 (4)	0.004 (4)	0.003 (3)	−0.006 (3)
C106	0.039 (5)	0.029 (5)	0.022 (4)	−0.004 (4)	−0.002 (3)	−0.003 (3)
C107	0.051 (6)	0.027 (5)	0.024 (4)	0.006 (4)	0.000 (3)	0.002 (3)
C108	0.042 (5)	0.028 (5)	0.022 (4)	−0.002 (4)	0.001 (4)	0.003 (3)
C11	0.018 (4)	0.024 (4)	0.021 (4)	0.000 (3)	0.000 (3)	−0.002 (3)
N12	0.025 (3)	0.023 (3)	0.017 (3)	−0.002 (3)	0.002 (3)	−0.002 (3)
C121	0.028 (4)	0.021 (4)	0.016 (4)	−0.001 (3)	−0.001 (3)	−0.001 (3)
C122	0.029 (4)	0.026 (4)	0.019 (4)	0.002 (3)	−0.001 (3)	0.003 (3)
C123	0.031 (4)	0.020 (4)	0.026 (4)	−0.002 (3)	−0.008 (3)	0.002 (3)
C124	0.035 (4)	0.019 (4)	0.022 (4)	0.004 (3)	−0.004 (3)	−0.002 (3)
C125	0.032 (4)	0.025 (4)	0.017 (4)	0.010 (3)	−0.003 (3)	0.000 (3)
C126	0.033 (4)	0.017 (4)	0.031 (4)	0.007 (4)	−0.002 (4)	−0.004 (3)
C127	0.028 (4)	0.023 (4)	0.023 (4)	0.005 (3)	0.001 (3)	−0.002 (3)
C128	0.026 (4)	0.025 (4)	0.029 (4)	0.006 (3)	−0.004 (3)	0.009 (3)
C129	0.026 (4)	0.022 (3)	0.020 (3)	0.003 (4)	0.000 (3)	−0.001 (3)
C120	0.022 (4)	0.018 (4)	0.021 (3)	0.003 (3)	−0.004 (3)	0.002 (3)
C161	0.025 (4)	0.023 (4)	0.021 (4)	−0.003 (3)	0.002 (3)	−0.003 (3)
C162	0.028 (4)	0.026 (4)	0.028 (4)	−0.003 (3)	−0.001 (4)	0.001 (4)
C163	0.029 (5)	0.033 (5)	0.036 (5)	−0.003 (4)	0.003 (4)	−0.002 (4)
C164	0.032 (5)	0.033 (5)	0.034 (5)	−0.004 (4)	0.008 (4)	−0.006 (4)
C165	0.038 (5)	0.039 (5)	0.025 (4)	−0.012 (4)	0.003 (4)	0.002 (4)
C166	0.034 (4)	0.031 (5)	0.020 (4)	−0.006 (4)	0.000 (3)	0.003 (3)
C171	0.031 (4)	0.029 (5)	0.026 (4)	−0.001 (4)	0.004 (3)	−0.002 (3)
C172	0.034 (5)	0.027 (4)	0.029 (4)	−0.002 (3)	0.001 (4)	0.003 (3)
C173	0.035 (4)	0.027 (4)	0.038 (4)	−0.001 (4)	0.002 (3)	0.004 (4)
C174	0.034 (5)	0.044 (6)	0.036 (5)	−0.006 (4)	0.007 (4)	0.000 (4)
C175	0.030 (5)	0.040 (6)	0.044 (5)	0.004 (4)	−0.002 (4)	−0.004 (4)
C176	0.031 (5)	0.026 (5)	0.048 (5)	0.004 (3)	0.004 (4)	0.001 (4)
C13	0.028 (4)	0.019 (4)	0.023 (4)	0.001 (3)	0.002 (3)	0.001 (3)
C14	0.024 (4)	0.023 (4)	0.017 (4)	−0.001 (3)	0.000 (3)	−0.001 (3)
N15	0.025 (3)	0.020 (3)	0.022 (3)	−0.001 (3)	−0.002 (3)	0.000 (3)
C151	0.030 (4)	0.019 (4)	0.022 (3)	0.001 (4)	−0.001 (3)	−0.001 (3)
C152	0.026 (4)	0.024 (4)	0.021 (3)	−0.002 (3)	−0.001 (3)	−0.001 (3)
C153	0.032 (4)	0.024 (4)	0.028 (4)	−0.004 (3)	0.000 (3)	−0.004 (3)
C154	0.043 (5)	0.023 (4)	0.022 (4)	0.004 (3)	−0.001 (4)	−0.005 (3)
C155	0.035 (5)	0.021 (4)	0.022 (4)	0.007 (3)	0.002 (3)	−0.002 (3)
C156	0.037 (5)	0.023 (4)	0.022 (4)	0.004 (3)	0.005 (3)	−0.001 (3)
C157	0.035 (5)	0.027 (4)	0.024 (4)	0.010 (4)	0.006 (3)	0.009 (3)
C158	0.029 (4)	0.027 (4)	0.018 (4)	0.002 (3)	−0.003 (3)	0.006 (3)
C159	0.035 (5)	0.019 (4)	0.022 (4)	0.005 (3)	0.001 (3)	0.000 (3)
C150	0.031 (4)	0.018 (4)	0.022 (4)	0.005 (3)	0.002 (3)	0.002 (3)
C181	0.032 (5)	0.022 (4)	0.025 (4)	−0.001 (3)	−0.001 (3)	−0.006 (3)
C182	0.031 (5)	0.026 (4)	0.035 (4)	−0.002 (3)	−0.003 (4)	0.004 (3)
C183	0.043 (5)	0.030 (5)	0.035 (5)	−0.005 (4)	0.004 (4)	0.000 (4)
C184	0.033 (5)	0.029 (5)	0.047 (5)	−0.003 (4)	0.004 (4)	−0.001 (4)
C185	0.026 (4)	0.042 (5)	0.035 (4)	0.000 (4)	−0.004 (3)	−0.003 (5)
C186	0.029 (4)	0.031 (5)	0.030 (4)	0.000 (3)	−0.005 (3)	0.000 (3)
C191	0.034 (5)	0.028 (5)	0.027 (4)	0.001 (3)	0.000 (3)	−0.005 (3)
C192	0.044 (6)	0.078 (8)	0.035 (5)	−0.015 (6)	−0.002 (4)	0.016 (6)
C193	0.038 (5)	0.097 (9)	0.037 (5)	−0.016 (6)	−0.011 (5)	0.027 (5)
C194	0.037 (5)	0.052 (6)	0.039 (5)	−0.003 (4)	−0.002 (4)	0.008 (5)
C195	0.035 (4)	0.031 (4)	0.038 (4)	0.002 (3)	0.005 (4)	0.007 (4)
C196	0.040 (5)	0.042 (6)	0.031 (4)	−0.001 (4)	0.002 (4)	0.013 (4)
C16	0.035 (4)	0.022 (4)	0.026 (4)	0.000 (3)	−0.005 (3)	−0.004 (3)
C17	0.038 (4)	0.029 (5)	0.024 (4)	0.001 (4)	−0.001 (3)	0.000 (4)
C18	0.034 (4)	0.018 (4)	0.031 (4)	0.002 (3)	−0.002 (3)	0.005 (3)
C19	0.038 (5)	0.020 (4)	0.021 (4)	0.002 (3)	−0.002 (3)	−0.004 (3)

### [µ2-(Sa,Sa,3aR,7aR)-1,3-bis(2,7-Dicyclohexylnaphthalen-1-yl)octahydro-1H-benzo[d]imidazolidin-2-ylidene]chlorido(η4-1,5-cyclooctadiene)iridium (II) Geometric parameters (Å, º)

**Table:**

Ir1—C11	2.045 (8)	C174—H17H	0.99
Ir1—C104	2.132 (8)	C175—C176	1.533 (13)
Ir1—C103	2.140 (8)	C175—H17I	0.99
Ir1—C102	2.181 (7)	C175—H17J	0.99
Ir1—C101	2.228 (7)	C176—H17K	0.99
Ir1—Cl1	2.3592 (18)	C176—H17L	0.99
C101—C102	1.405 (13)	C13—C14	1.496 (11)
C101—C106	1.515 (12)	C13—C16	1.502 (12)
C101—H101	0.95	C13—H13	1
C102—C107	1.507 (13)	C14—N15	1.479 (10)
C102—H102	0.95	C14—C19	1.504 (11)
C103—C104	1.435 (12)	C14—H14	1
C103—C108	1.508 (12)	N15—C151	1.443 (10)
C103—H103	0.95	C151—C152	1.377 (11)
C104—C105	1.501 (12)	C151—C150	1.429 (11)
C104—H104	0.95	C152—C153	1.433 (12)
C105—C106	1.514 (12)	C152—C181	1.506 (11)
C105—H10A	0.99	C153—C154	1.366 (13)
C105—H10B	0.99	C153—H153	0.95
C106—H10C	0.99	C154—C155	1.393 (13)
C106—H10D	0.99	C154—H154	0.95
C107—C108	1.536 (13)	C155—C156	1.408 (12)
C107—H10E	0.99	C155—C150	1.431 (11)
C107—H10F	0.99	C156—C157	1.373 (13)
C108—H10G	0.99	C156—H156	0.95
C108—H10H	0.99	C157—C158	1.403 (12)
C11—N15	1.339 (11)	C157—H157	0.95
C11—N12	1.363 (10)	C158—C159	1.372 (12)
N12—C121	1.449 (10)	C158—C191	1.503 (12)
N12—C13	1.478 (10)	C159—C150	1.417 (12)
C121—C122	1.374 (12)	C159—H159	0.95
C121—C120	1.433 (11)	C181—C186	1.538 (12)
C122—C123	1.427 (12)	C181—C182	1.539 (12)
C122—C161	1.511 (11)	C181—H181	1
C123—C124	1.356 (12)	C182—C183	1.524 (13)
C123—H123	0.95	C182—H18A	0.99
C124—C125	1.408 (12)	C182—H18B	0.99
C124—H124	0.95	C183—C184	1.535 (14)
C125—C120	1.413 (11)	C183—H18C	0.99
C125—C126	1.430 (12)	C183—H18D	0.99
C126—C127	1.370 (13)	C184—C185	1.525 (14)
C126—H126	0.95	C184—H18E	0.99
C127—C128	1.417 (12)	C184—H18F	0.99
C127—H127	0.95	C185—C186	1.547 (12)
C128—C129	1.372 (11)	C185—H18G	0.99
C128—C171	1.505 (12)	C185—H18H	0.99
C129—C120	1.426 (11)	C186—H18I	0.99
C129—H129	0.95	C186—H18J	0.99
C161—C166	1.524 (11)	C191—C196	1.535 (13)
C161—C162	1.543 (12)	C191—C192	1.550 (15)
C161—H161	1	C191—H191	1
C162—C163	1.532 (12)	C192—C193	1.515 (15)
C162—H16A	0.99	C192—H19A	0.99
C162—H16B	0.99	C192—H19B	0.99
C163—C164	1.522 (13)	C193—C194	1.526 (15)
C163—H16C	0.99	C193—H19C	0.99
C163—H16D	0.99	C193—H19D	0.99
C164—C165	1.526 (13)	C194—C195	1.510 (14)
C164—H16E	0.99	C194—H19E	0.99
C164—H16F	0.99	C194—H19F	0.99
C165—C166	1.534 (13)	C195—C196	1.529 (14)
C165—H16G	0.99	C195—H19G	0.99
C165—H16H	0.99	C195—H19H	0.99
C166—H16I	0.99	C196—H19I	0.99
C166—H16J	0.99	C196—H19J	0.99
C171—C172	1.526 (12)	C16—C17	1.543 (12)
C171—C176	1.529 (13)	C16—H16K	0.99
C171—H171	1	C16—H16L	0.99
C172—C173	1.528 (12)	C17—C18	1.525 (12)
C172—H17C	0.99	C17—H17A	0.99
C172—H17D	0.99	C17—H17B	0.99
C173—C174	1.523 (13)	C18—C19	1.547 (11)
C173—H17E	0.99	C18—H18K	0.99
C173—H17F	0.99	C18—H18L	0.99
C174—C175	1.513 (14)	C19—H19K	0.99
C174—H17G	0.99	C19—H19L	0.99
			
C11—Ir1—C104	95.4 (3)	C175—C174—H17H	109.3
C11—Ir1—C103	97.2 (3)	C173—C174—H17H	109.3
C104—Ir1—C103	39.3 (3)	H17G—C174—H17H	107.9
C11—Ir1—C102	156.0 (3)	C174—C175—C176	111.1 (8)
C104—Ir1—C102	97.9 (3)	C174—C175—H17I	109.4
C103—Ir1—C102	80.9 (3)	C176—C175—H17I	109.4
C11—Ir1—C101	166.6 (3)	C174—C175—H17J	109.4
C104—Ir1—C101	80.4 (3)	C176—C175—H17J	109.4
C103—Ir1—C101	87.7 (3)	H17I—C175—H17J	108
C102—Ir1—C101	37.2 (3)	C171—C176—C175	112.1 (8)
C11—Ir1—Cl1	88.6 (2)	C171—C176—H17K	109.2
C104—Ir1—Cl1	153.7 (2)	C175—C176—H17K	109.2
C103—Ir1—Cl1	165.4 (2)	C171—C176—H17L	109.2
C102—Ir1—Cl1	88.3 (2)	C175—C176—H17L	109.2
C101—Ir1—Cl1	89.7 (2)	H17K—C176—H17L	107.9
C102—C101—C106	126.8 (8)	N12—C13—C14	100.7 (6)
C102—C101—Ir1	69.6 (4)	N12—C13—C16	122.2 (7)
C106—C101—Ir1	111.3 (5)	C14—C13—C16	111.0 (7)
C102—C101—H101	116.6	N12—C13—H13	107.4
C106—C101—H101	116.6	C14—C13—H13	107.4
Ir1—C101—H101	89	C16—C13—H13	107.4
C101—C102—C107	126.4 (8)	N15—C14—C13	100.0 (6)
C101—C102—Ir1	73.2 (4)	N15—C14—C19	121.3 (7)
C107—C102—Ir1	107.1 (5)	C13—C14—C19	111.5 (6)
C101—C102—H102	116.8	N15—C14—H14	107.8
C107—C102—H102	116.8	C13—C14—H14	107.8
Ir1—C102—H102	89.6	C19—C14—H14	107.8
C104—C103—C108	124.6 (7)	C11—N15—C151	129.0 (7)
C104—C103—Ir1	70.1 (5)	C11—N15—C14	111.3 (6)
C108—C103—Ir1	113.6 (6)	C151—N15—C14	118.0 (6)
C104—C103—H103	117.7	C152—C151—C150	122.2 (7)
C108—C103—H103	117.7	C152—C151—N15	120.3 (7)
Ir1—C103—H103	86.3	C150—C151—N15	117.2 (7)
C103—C104—C105	124.9 (8)	C151—C152—C153	117.5 (7)
C103—C104—Ir1	70.7 (5)	C151—C152—C181	124.4 (7)
C105—C104—Ir1	110.6 (6)	C153—C152—C181	118.0 (7)
C103—C104—H104	117.5	C154—C153—C152	121.5 (8)
C105—C104—H104	117.5	C154—C153—H153	119.2
Ir1—C104—H104	88.8	C152—C153—H153	119.2
C104—C105—C106	113.9 (7)	C153—C154—C155	121.5 (8)
C104—C105—H10A	108.8	C153—C154—H154	119.3
C106—C105—H10A	108.8	C155—C154—H154	119.3
C104—C105—H10B	108.8	C154—C155—C156	122.5 (7)
C106—C105—H10B	108.8	C154—C155—C150	119.0 (8)
H10A—C105—H10B	107.7	C156—C155—C150	118.5 (8)
C105—C106—C101	112.6 (7)	C157—C156—C155	121.4 (8)
C105—C106—H10C	109.1	C157—C156—H156	119.3
C101—C106—H10C	109.1	C155—C156—H156	119.3
C105—C106—H10D	109.1	C156—C157—C158	120.9 (8)
C101—C106—H10D	109.1	C156—C157—H157	119.5
H10C—C106—H10D	107.8	C158—C157—H157	119.5
C102—C107—C108	112.2 (7)	C159—C158—C157	118.5 (8)
C102—C107—H10E	109.2	C159—C158—C191	120.1 (7)
C108—C107—H10E	109.2	C157—C158—C191	121.2 (7)
C102—C107—H10F	109.2	C158—C159—C150	122.8 (7)
C108—C107—H10F	109.2	C158—C159—H159	118.6
H10E—C107—H10F	107.9	C150—C159—H159	118.6
C103—C108—C107	110.9 (7)	C159—C150—C151	124.0 (7)
C103—C108—H10G	109.5	C159—C150—C155	117.7 (7)
C107—C108—H10G	109.5	C151—C150—C155	118.2 (7)
C103—C108—H10H	109.5	C152—C181—C186	111.9 (7)
C107—C108—H10H	109.5	C152—C181—C182	110.8 (7)
H10G—C108—H10H	108.1	C186—C181—C182	110.1 (7)
N15—C11—N12	107.2 (7)	C152—C181—H181	108
N15—C11—Ir1	126.9 (6)	C186—C181—H181	108
N12—C11—Ir1	125.5 (6)	C182—C181—H181	108
C11—N12—C121	129.1 (7)	C183—C182—C181	111.9 (7)
C11—N12—C13	109.5 (6)	C183—C182—H18A	109.2
C121—N12—C13	120.5 (6)	C181—C182—H18A	109.2
C122—C121—C120	121.0 (7)	C183—C182—H18B	109.2
C122—C121—N12	120.6 (7)	C181—C182—H18B	109.2
C120—C121—N12	117.4 (7)	H18A—C182—H18B	107.9
C121—C122—C123	118.3 (8)	C182—C183—C184	111.2 (8)
C121—C122—C161	123.5 (7)	C182—C183—H18C	109.4
C123—C122—C161	118.0 (7)	C184—C183—H18C	109.4
C124—C123—C122	121.7 (8)	C182—C183—H18D	109.4
C124—C123—H123	119.2	C184—C183—H18D	109.4
C122—C123—H123	119.2	H18C—C183—H18D	108
C123—C124—C125	120.5 (7)	C185—C184—C183	110.1 (8)
C123—C124—H124	119.8	C185—C184—H18E	109.6
C125—C124—H124	119.8	C183—C184—H18E	109.6
C124—C125—C120	119.3 (7)	C185—C184—H18F	109.6
C124—C125—C126	122.3 (7)	C183—C184—H18F	109.6
C120—C125—C126	118.3 (7)	H18E—C184—H18F	108.2
C127—C126—C125	121.1 (7)	C184—C185—C186	110.7 (8)
C127—C126—H126	119.4	C184—C185—H18G	109.5
C125—C126—H126	119.4	C186—C185—H18G	109.5
C126—C127—C128	121.1 (7)	C184—C185—H18H	109.5
C126—C127—H127	119.4	C186—C185—H18H	109.5
C128—C127—H127	119.4	H18G—C185—H18H	108.1
C129—C128—C127	118.2 (8)	C181—C186—C185	111.2 (7)
C129—C128—C171	122.3 (8)	C181—C186—H18I	109.4
C127—C128—C171	119.5 (7)	C185—C186—H18I	109.4
C128—C129—C120	122.6 (7)	C181—C186—H18J	109.4
C128—C129—H129	118.7	C185—C186—H18J	109.4
C120—C129—H129	118.7	H18I—C186—H18J	108
C125—C120—C129	118.5 (7)	C158—C191—C196	115.1 (7)
C125—C120—C121	118.7 (7)	C158—C191—C192	109.3 (8)
C129—C120—C121	122.7 (7)	C196—C191—C192	108.6 (8)
C122—C161—C166	114.4 (7)	C158—C191—H191	107.8
C122—C161—C162	110.4 (6)	C196—C191—H191	107.8
C166—C161—C162	108.4 (7)	C192—C191—H191	107.8
C122—C161—H161	107.8	C193—C192—C191	113.0 (10)
C166—C161—H161	107.8	C193—C192—H19A	109
C162—C161—H161	107.8	C191—C192—H19A	109
C163—C162—C161	110.4 (7)	C193—C192—H19B	109
C163—C162—H16A	109.6	C191—C192—H19B	109
C161—C162—H16A	109.6	H19A—C192—H19B	107.8
C163—C162—H16B	109.6	C192—C193—C194	111.1 (8)
C161—C162—H16B	109.6	C192—C193—H19C	109.4
H16A—C162—H16B	108.1	C194—C193—H19C	109.4
C164—C163—C162	112.3 (8)	C192—C193—H19D	109.4
C164—C163—H16C	109.1	C194—C193—H19D	109.4
C162—C163—H16C	109.1	H19C—C193—H19D	108
C164—C163—H16D	109.1	C195—C194—C193	111.2 (9)
C162—C163—H16D	109.1	C195—C194—H19E	109.4
H16C—C163—H16D	107.9	C193—C194—H19E	109.4
C163—C164—C165	112.7 (7)	C195—C194—H19F	109.4
C163—C164—H16E	109.1	C193—C194—H19F	109.4
C165—C164—H16E	109.1	H19E—C194—H19F	108
C163—C164—H16F	109.1	C194—C195—C196	111.4 (8)
C165—C164—H16F	109.1	C194—C195—H19G	109.4
H16E—C164—H16F	107.8	C196—C195—H19G	109.4
C164—C165—C166	111.0 (7)	C194—C195—H19H	109.4
C164—C165—H16G	109.4	C196—C195—H19H	109.4
C166—C165—H16G	109.4	H19G—C195—H19H	108
C164—C165—H16H	109.4	C195—C196—C191	112.5 (8)
C166—C165—H16H	109.4	C195—C196—H19I	109.1
H16G—C165—H16H	108	C191—C196—H19I	109.1
C161—C166—C165	110.6 (7)	C195—C196—H19J	109.1
C161—C166—H16I	109.5	C191—C196—H19J	109.1
C165—C166—H16I	109.5	H19I—C196—H19J	107.8
C161—C166—H16J	109.5	C13—C16—C17	106.3 (7)
C165—C166—H16J	109.5	C13—C16—H16K	110.5
H16I—C166—H16J	108.1	C17—C16—H16K	110.5
C128—C171—C172	114.4 (7)	C13—C16—H16L	110.5
C128—C171—C176	110.9 (7)	C17—C16—H16L	110.5
C172—C171—C176	109.6 (7)	H16K—C16—H16L	108.7
C128—C171—H171	107.2	C18—C17—C16	113.5 (7)
C172—C171—H171	107.2	C18—C17—H17A	108.9
C176—C171—H171	107.2	C16—C17—H17A	108.9
C171—C172—C173	111.7 (7)	C18—C17—H17B	108.9
C171—C172—H17C	109.3	C16—C17—H17B	108.9
C173—C172—H17C	109.3	H17A—C17—H17B	107.7
C171—C172—H17D	109.3	C17—C18—C19	113.4 (7)
C173—C172—H17D	109.3	C17—C18—H18K	108.9
H17C—C172—H17D	107.9	C19—C18—H18K	108.9
C174—C173—C172	110.2 (8)	C17—C18—H18L	108.9
C174—C173—H17E	109.6	C19—C18—H18L	108.9
C172—C173—H17E	109.6	H18K—C18—H18L	107.7
C174—C173—H17F	109.6	C14—C19—C18	105.4 (7)
C172—C173—H17F	109.6	C14—C19—H19K	110.7
H17E—C173—H17F	108.1	C18—C19—H19K	110.7
C175—C174—C173	111.7 (8)	C14—C19—H19L	110.7
C175—C174—H17G	109.3	C18—C19—H19L	110.7
C173—C174—H17G	109.3	H19K—C19—H19L	108.8
			
C106—C101—C102—C107	−2.7 (13)	C11—N12—C13—C16	153.0 (7)
Ir1—C101—C102—C107	99.2 (8)	C121—N12—C13—C16	−37.0 (11)
C106—C101—C102—Ir1	−101.9 (8)	N12—C13—C14—N15	−31.5 (7)
C108—C103—C104—C105	−3.5 (13)	C16—C13—C14—N15	−162.2 (6)
Ir1—C103—C104—C105	102.1 (8)	N12—C13—C14—C19	−161.0 (6)
C108—C103—C104—Ir1	−105.5 (8)	C16—C13—C14—C19	68.3 (9)
C103—C104—C105—C106	−42.6 (12)	N12—C11—N15—C151	−173.3 (7)
Ir1—C104—C105—C106	37.6 (9)	Ir1—C11—N15—C151	13.5 (12)
C104—C105—C106—C101	−32.9 (11)	N12—C11—N15—C14	−8.7 (9)
C102—C101—C106—C105	92.2 (10)	Ir1—C11—N15—C14	178.0 (5)
Ir1—C101—C106—C105	12.3 (9)	C13—C14—N15—C11	26.6 (8)
C101—C102—C107—C108	−39.2 (11)	C19—C14—N15—C11	149.5 (7)
Ir1—C102—C107—C108	42.3 (8)	C13—C14—N15—C151	−167.0 (6)
C104—C103—C108—C107	97.0 (10)	C19—C14—N15—C151	−44.1 (10)
Ir1—C103—C108—C107	15.7 (9)	C11—N15—C151—C152	−87.1 (11)
C102—C107—C108—C103	−39.0 (10)	C14—N15—C151—C152	109.3 (9)
N15—C11—N12—C121	177.3 (7)	C11—N15—C151—C150	97.8 (10)
Ir1—C11—N12—C121	−9.3 (12)	C14—N15—C151—C150	−65.9 (9)
N15—C11—N12—C13	−13.8 (9)	C150—C151—C152—C153	0.9 (12)
Ir1—C11—N12—C13	159.5 (5)	N15—C151—C152—C153	−174.0 (7)
C11—N12—C121—C122	−83.8 (11)	C150—C151—C152—C181	177.1 (7)
C13—N12—C121—C122	108.3 (8)	N15—C151—C152—C181	2.2 (12)
C11—N12—C121—C120	107.1 (9)	C151—C152—C153—C154	0.6 (12)
C13—N12—C121—C120	−60.8 (10)	C181—C152—C153—C154	−175.9 (8)
C120—C121—C122—C123	−4.7 (11)	C152—C153—C154—C155	−0.6 (13)
N12—C121—C122—C123	−173.5 (7)	C153—C154—C155—C156	179.2 (8)
C120—C121—C122—C161	170.1 (7)	C153—C154—C155—C150	−0.9 (13)
N12—C121—C122—C161	1.4 (11)	C154—C155—C156—C157	−178.3 (8)
C121—C122—C123—C124	6.9 (11)	C150—C155—C156—C157	1.8 (12)
C161—C122—C123—C124	−168.2 (7)	C155—C156—C157—C158	−3.1 (12)
C122—C123—C124—C125	−2.5 (12)	C156—C157—C158—C159	0.9 (12)
C123—C124—C125—C120	−4.1 (11)	C156—C157—C158—C191	176.1 (8)
C123—C124—C125—C126	175.3 (7)	C157—C158—C159—C150	2.6 (12)
C124—C125—C126—C127	−179.3 (8)	C191—C158—C159—C150	−172.6 (7)
C120—C125—C126—C127	0.1 (11)	C158—C159—C150—C151	175.5 (8)
C125—C126—C127—C128	−2.9 (12)	C158—C159—C150—C155	−3.8 (11)
C126—C127—C128—C129	2.1 (12)	C152—C151—C150—C159	178.3 (8)
C126—C127—C128—C171	−176.2 (8)	N15—C151—C150—C159	−6.6 (11)
C127—C128—C129—C120	1.4 (11)	C152—C151—C150—C155	−2.3 (12)
C171—C128—C129—C120	179.7 (7)	N15—C151—C150—C155	172.7 (7)
C124—C125—C120—C129	−177.3 (7)	C154—C155—C150—C159	−178.3 (8)
C126—C125—C120—C129	3.2 (10)	C156—C155—C150—C159	1.6 (11)
C124—C125—C120—C121	6.1 (10)	C154—C155—C150—C151	2.3 (11)
C126—C125—C120—C121	−173.4 (7)	C156—C155—C150—C151	−177.8 (7)
C128—C129—C120—C125	−4.0 (11)	C151—C152—C181—C186	122.8 (8)
C128—C129—C120—C121	172.4 (7)	C153—C152—C181—C186	−61.0 (10)
C122—C121—C120—C125	−1.6 (11)	C151—C152—C181—C182	−113.9 (9)
N12—C121—C120—C125	167.5 (7)	C153—C152—C181—C182	62.3 (9)
C122—C121—C120—C129	−178.0 (7)	C152—C181—C182—C183	−179.2 (7)
N12—C121—C120—C129	−9.0 (10)	C186—C181—C182—C183	−54.9 (9)
C121—C122—C161—C166	131.4 (8)	C181—C182—C183—C184	56.4 (10)
C123—C122—C161—C166	−53.7 (10)	C182—C183—C184—C185	−57.3 (10)
C121—C122—C161—C162	−106.0 (8)	C183—C184—C185—C186	57.5 (10)
C123—C122—C161—C162	68.8 (9)	C152—C181—C186—C185	178.7 (7)
C122—C161—C162—C163	174.2 (7)	C182—C181—C186—C185	55.0 (9)
C166—C161—C162—C163	−59.9 (9)	C184—C185—C186—C181	−57.3 (10)
C161—C162—C163—C164	54.9 (9)	C159—C158—C191—C196	−136.9 (8)
C162—C163—C164—C165	−50.8 (10)	C157—C158—C191—C196	48.0 (11)
C163—C164—C165—C166	51.6 (10)	C159—C158—C191—C192	100.5 (9)
C122—C161—C166—C165	−174.7 (7)	C157—C158—C191—C192	−74.5 (10)
C162—C161—C166—C165	61.6 (9)	C158—C191—C192—C193	−179.2 (9)
C164—C165—C166—C161	−57.7 (10)	C196—C191—C192—C193	54.5 (12)
C129—C128—C171—C172	26.9 (11)	C191—C192—C193—C194	−55.7 (15)
C127—C128—C171—C172	−154.8 (7)	C192—C193—C194—C195	55.3 (14)
C129—C128—C171—C176	−97.7 (9)	C193—C194—C195—C196	−55.6 (12)
C127—C128—C171—C176	80.6 (9)	C194—C195—C196—C191	56.4 (11)
C128—C171—C172—C173	178.1 (7)	C158—C191—C196—C195	−177.2 (8)
C176—C171—C172—C173	−56.7 (9)	C192—C191—C196—C195	−54.3 (11)
C171—C172—C173—C174	57.3 (10)	N12—C13—C16—C17	−177.9 (7)
C172—C173—C174—C175	−56.1 (10)	C14—C13—C16—C17	−59.4 (9)
C173—C174—C175—C176	54.9 (10)	C13—C16—C17—C18	52.6 (10)
C128—C171—C176—C175	−177.5 (7)	C16—C17—C18—C19	−52.6 (10)
C172—C171—C176—C175	55.2 (10)	N15—C14—C19—C18	−178.1 (7)
C174—C175—C176—C171	−54.8 (11)	C13—C14—C19—C18	−60.9 (9)
C11—N12—C13—C14	29.7 (8)	C17—C18—C19—C14	53.5 (9)
C121—N12—C13—C14	−160.3 (7)		
